# Geometry-conditioned diffusion generation of Lagrangian particle trajectories in human airways

**DOI:** 10.21203/rs.3.rs-10503600/v1

**Published:** 2026-08-04

**Authors:** Ignacio R. Bartol, Martin Graffigna Palomba, Mauricio E. Tano, Shaheen A. Dewji

**Affiliations:** a Nuclear and Radiological Engineering and Medical Physics Program, George W. Woodruff School of Mechanical Engineering, Georgia Institute of Technology, 770 State St NW, Atlanta, 30332-0405, GA, United States of America; b Collaborative Computing Center (C3), Idaho National Laboratory, 955 MK Simpson Boulevard, Idaho Falls, 83415, ID, United States of America

**Keywords:** Lagrangian particle tracking, human airways, diffusion probabilistic model, geometry conditioning, signed distance function, aerosol transport

## Abstract

Fast and subject-specific prediction of particle transport in the human airways is central to quantifying aerosol deposition, optimizing inhaled drug delivery, and supporting dose assessment in radiation research. While computational fluid and particle dynamics simulations provide high-fidelity trajectory data, their use in clinical or large-cohort settings remains limited by stringent geometric preprocessing (segmentation quality control, topology repair, surface smoothing, and meshing) and by the high computational cost of coupled airflow-particle tracking. Recent data-driven surrogates have accelerated aspects of airway flow prediction, but most approaches focus on Eulerian flow fields, operate in simplified or two-dimensional settings, and often struggle to generalize across complex three-dimensional anatomies. Especially for particle trajectories in a Lagrangian frame of reference, which are intrinsically multimodal and sensitive to local geometry.

In this work we propose a geometry-conditioned *guided denoising diffusion probabilistic model* that generates physically plausible particle trajectories directly in a Lagrangian representation. Airway anatomy is encoded by a variational autoencoder to provide a compact conditioning latent, and geometric feasibility during sampling is reinforced through signed distance function guidance that discourages samples leaving the airway lumen. Despite training on particle-track data from only nine simulated airway geometries, the proposed method produces trajectory ensembles that qualitatively match ground-truth branching patterns and recover the underlying velocity magnitude statistics with good agreement, while guidance reduces non-physical wall-penetrating outliers. Remaining limitations include occasional boundary violations in challenging cases, reduced diversity in some distal branches under strong guidance, and degraded behavior for out-of-distribution geometries not represented by the limited training set.

## 1. Introduction

Inhaled particle transport in the human respiratory tract spans a broad range of highly sensitive applications, from radioactive aerosol exposure assessment and respiratory toxicology to the design and optimization of inhaled therapeutics. For medical aerosols, therapeutic effectiveness depends on where and how much of the delivered dose deposits across the extrathoracic region, conducting airways, and peripheral lung, which is influenced by airway morphology, breathing waveform, and particle size distribution Labiris and Dolovich (2003); Longest and Holbrook (2012); Kundu and Panchagnula (2025); Kleinstreuer and Zhang (2010). As a result, subject-specific predictions are attractive for patient-tailored drug delivery and for identifying inter-subject variability in regional deposition Poorbahrami et al. (2019); Huang et al. (2021).

Beyond pharmacological aerosols, detailed particle tracking is central to inhalation dosimetry for radioactive aerosols found in occupational, environmental, and radiological accident scenarios ICRP (1994); Talaat et al. (2019); Bartol et al. (2024b). While regulatory and radiological protection frameworks often rely on population-average deposition models ICRP (1994), several studies have shown that high-resolution computational predictions can localize deposition hot spots and translate them into spatially resolved internal dose estimates that traditional models would have missed assuming uniform deposition across the airway lumen Talaat et al. (2019); Poorbahrami et al. (2019); Bartol et al. (2024b).

Nonetheless, modeling these aerosols is challenging. A multiphysics modeling approach is required; particle deposition is influenced by the interplay of inertial impaction, gravitational sedimentation, and Brownian diffusion, with the relative importance of each mechanism varying strongly with particle size, airway region, and local flow structures Darquenne (2014); Hofmann (2011); Kleinstreuer and Zhang (2010). Consequently, high-fidelity simulations using Computational Fluid and Particle Dynamics (CFPD) remain too time-consuming for many clinical workflows that require rapid exploration of inhaler settings, breathing conditions, or patient anatomies.

State-of-the-art CFPD typically solves airflow using the Navier-Stokes equation in an Eulerian frame of reference, and tracks particles using a Lagrangian formulation, providing highly detailed, spatially resolved predictions of transport and particle deposition profiles Nowak et al. (2003); Longest and Holbrook (2012); Rostami (2009). Despite its accuracy and interpretability, constructing a subject- and scenario-specific CFPD pipeline is non-trivial. The workflow requires: segmentation of the airway tree from a medical imaging (computed tomography or magnetic resonance), 3D surface reconstruction and its repair (e.g., removal of disconnected branches, correction of holes and non-manifold regions), generation of a high-quality volumetric mesh, and careful specification of boundary conditions and turbulence modeling assumptions Longest and Holbrook (2012); Huang et al. (2021); Longest et al. (2019); Bartol et al. (2025). These steps are often stringent because small geometric defects (particularly near bronchi bifurcations) can produce ill-conditioned meshes or non-physical flow artifacts that propagate into particle deposition predictions Huang et al. (2021); Longest et al. (2019).

Even after preprocessing steps, computational wall time remains the primary bottleneck for computing a diverse dataset that covers multiple boundary conditions. High-fidelity predictions may require transient simulations, fine near-wall resolution, and large particle datasets to reduce variance and resolve rare events in the tail of deposition statistics Kleinstreuer and Zhang (2010); Longest et al. (2019); Huang et al. (2021). Consequently, while CFPD is well-suited for detailed analysis and device development, it is challenging to deploy at scale across large cohorts or embed in iterative clinical decision support.

Motivated by the cost of numerical simulation, machine learning surrogates for fluid dynamics have grown rapidly Brunton et al. (2020); Kochkov et al. (2021); Kutz and Brunton (2016). Early approaches using deep learning often encode geometry into a fixed grid and perform regression on Eulerian flow observables such as velocity or pressure fields Guo et al. (2016). More recent operator-learning approaches aim to learn mappings between function spaces, which are considered more interpretable and can make predictions across families of partial differential equations Lu et al. (2021); Li et al. (2021); Huhn et al. (2023); Graffigna et al. (2026). Another approach used mesh-based graph neural networks, which provided a route to learning directly on unstructured discretizations Pfaff et al. (2021) and have the potential to overcome the limitation of other DL methods that worked only on structured grids. These advances meaningfully expand what is possible with data-driven models for computational physics Brunton et al. (2020); Kochkov et al. (2021), but they have been demonstrated only for Eulerian flow-field prediction, with limited translation to Lagrangian formulations.

Several gaps remain for subject-specific airway particle tracking. First, many learning-based surrogates are evaluated on simplified benchmarks or on problems with controlled geometry parameterizations, leaving open questions about robustness on heterogeneous, patient-derived three-dimensional airway topologies Brunton et al. (2020); Guo et al. (2016); Lu et al. (2021); Graffigna et al. (2026). Second, the dominant target in the literature is the Eulerian carrier-phase field (or aggregated deposition metrics), whereas the downstream quantity of interest in inhalation studies is often the particle-by-particle deposition profile and its uncertainty, including the exact distribution of particle tracks, residence times, and deposition outcomes Longest and Holbrook (2012); Huang et al. (2021); Graffigna et al. (2026). As explained, trajectory generation is intrinsically complex and produces high-variance distributions; even with fixed boundary conditions, the ensemble variability produced by turbulence, near-wall interactions, and stochastic forces (relevant for submicron particles) could be different for the same geometry, and the spatially accurate prediction is critical for risk assessment and dose estimation Darquenne (2014); Hofmann (2011). Finally, purely deterministic regressors as in Han et al. (2022) do not naturally provide a generative mechanism for sampling multiple plausible trajectory realizations conditioned on patient geometry and inhalation settings.

Diffusion probabilistic models address conditional generative modeling by learning a gradual denoising process that maps Gaussian noise to data Sohl-Dickstein et al. (2015); Ho et al. (2020); Nichol and Dhariwal (2021). In the denoising diffusion probabilistic model formulation, a forward Markov chain progressively perturbs data with noise, while a neural network learns the reverse-time denoising transitions Ho et al. (2020); Nichol and Dhariwal (2021). A common parameterization trains a model to predict the injected noise at diffusion step t as in Eq. 1, where c denotes conditioning information Ho et al. (2020); Nichol and Dhariwal (2021).


(1) $$x_{t}=\sqrt{{\overset{‾}{\alpha }}_{t}}x_{0}+\sqrt{1-{\overset{‾}{\alpha }}_{t}}\epsilon ,\;\epsilon \sim 𝒩(0,I),\;ℒ=E\left[{\left\|\epsilon -\epsilon_{\theta }\left(x_{t},t,c\right)\right\|}_{2}^{2}\right],$$

Compared to previous generative models, generative adversarial networks (GANs) Goodfellow et al. (2020), diffusion models are widely recognized for stable training (a supervised denoising objective rather than adversarial minimax optimization) and strong empirical sample quality; as it is well known, diffusion models have been shown to exceed state-of-the-art GANs on standard image synthesis benchmarks Dhariwal and Nichol (2021).

Notably, base DDPM models would not suffice to bridge the gap in subject specificity. Conditioning of DDPMs is of utmost importance in this setting, where the experiment requires samples consistent with known context, which for this work is the airway anatomy. Modern conditional diffusion models incorporate rich conditioning pathways, such as cross-attention modules and learned embeddings Rombach et al. (2022); Liu et al. (2023b). Moreover, guided diffusion enables steering of samples at inference time to enforce constraints without retraining the base model, particularly in medical or physical imaging settings, where hallucinations play a vital role Liu et al. (2023a). In image synthesis, guidance has been implemented using auxiliary gradients (classifier guidance) Dhariwal and Nichol (2021) and by combining conditional and unconditional scores, both of which are commonly used in practice and have been formalized in latent diffusion frameworks Rombach et al. (2022). These ideas lay the groundwork for subject-specific trajectory generation, where airway geometric plausibility can be viewed as a constraint that is otherwise difficult to guarantee from data alone (i.e., only particle trajectories).

Recent evidence also indicates that diffusion models are well-suited to learning Lagrangian particle trajectory statistics in complex dynamical systems: diffusion-based generative modeling has been shown to reproduce multi-scale statistics of three-dimensional turbulent single-particle trajectories, including heavy tails and intermittency effects Li et al. (2024a). This motivates exploring diffusion models as samplers for physically plausible trajectory ensembles beyond the turbulence setting, provided that appropriate conditioning and constraints are incorporated.

In this paper, we propose a geometry-conditioned guided DDPM for fast generation of airway particle trajectories. The overarching goal of this work is to replace repeated, computationally costly simulation-based trajectory generation with a learned conditional model that can produce plausible particle tracks for a given airway geometry in seconds to minutes of neural inference, while retaining key statistical-physical features of the underlying dynamics.

The proposed approach combines three ideas. First, we learn a compact latent representation of patient airway geometry with a *variational encoder* Kingma and Welling (2014) and use this latent vector as conditioning input to the denoising diffusion probabilistic model. Second, we represent airway shape using a signed distance function field – a representation that provides an explicit notion of walls and smooth geometric gradients Park et al. (2019); Mescheder et al. (2019). Third, during inference, we apply *signed distance function guidance* to discourage samples that violate geometric feasibility (e.g., trajectories leaving the airway lumen), analogously to guidance mechanisms used in conditional diffusion Dhariwal and Nichol (2021); Rombach et al. (2022).

Our contributions in this work are summarized as follows:

1. a diffusion-based generative model for trajectory ensembles conditioned on three-dimensional patient geometry,
2. a variational encoding strategy that enables conditioning on anatomies not seen during training,
3. an inference-time guidance mechanism based on signed distance function geometry that improves geometric plausibility,
4. an evaluation demonstrating that the model can recover velocity magnitude statistics and produce visually consistent trajectory bundles, while also revealing that generalization to unseen anatomies remains fundamentally limited by the diversity of available simulated geometries.

## 2. Methods

### 2.1. Study overview and scope

This study used high-fidelity simulation-derived particle tracks in a Lagrangian framework as the reference data for training and evaluation of a geometry-conditioned DDPM. The input anatomy for conditioningwas represented as a 3D voxelized airway occupancy grid. The DDPM output was one fixed-length sequence of 3D particle positions per generated trajectory (i.e. a sequence of particle tracks). The airway anatomy grid was encoded by a variational geometry encoder (VAE) and supplied to the temporal denoiser as global anatomical conditioning. A signed distance field (SDF), constructed from the same voxelized geometry but not used as a learned conditioning input, was queried during sampling to provide a soft geometric penalty for out-of-lumen positions at inference time.

The inlet flow, particle-size distribution, injection protocol, airway-simulation settings, and terminal handling rules were inherited from the simulation dataset rather than independently conditioned at inference. Therefore, the implementation reported in this manuscript approximates a conditional distribution of position sequences given anatomy under a fixed data-generation regime and preprocessing regime represented in the training data. In its current verstion, it does not output carrier-flow variables, or a deposition/escape label. Comparisons with simulation-derived trajectories were made using the qualitative trajectory overlays and trajectory-derived kinematic metrics defined later in this section. In contrast to diffusion-based trajectory generators developed for unbounded homogeneous isotropic turbulence Li et al. (2024b), the present setting involves bounded, heterogeneous, subject-specific airway domains, which is a major challenge in real-world applications.

This separation between geometry representation, conditioning, and guidance is important for model interpretability. In one hand, the occupancy grid supplies a learned global geometric embedding, whereas the SDF is a directly queried geometric object to correct particle trajectories used during inference. The reference particle-diameter distribution, injection parameters, and breathing conditions (waveform, intensity) defined the regime from which trajectories were sampled; in this study they were not separate conditioning variables for the DDPM as mentioned before. As a result, evaluation focuses on whether generated position sequences follow the represented airway topology and reproduce a broad set of physical metrics focused on particle kinematics.

The remainder of this section describes the reference trajectory source, trajectory representation, geometric preprocessing, conditional diffusion model, SDF-guided sampling procedure, architecture and training protocol, metric definitions, and how we used dynamic mode decomposition (DMD) as baseline to compare differet surrogate models. The carrier-phase and Lagrangian simulation information are included only facilitate the reading flow and provide context for the fixed reference-data regime, the details on the simulation settings can be found in previous work from Bartol et al. (2025, 2024b).

### 2.2. Reference trajectory dataset

#### 2.2.1. Large-cohort airway segmentation and representative downselection

Airway geometries were reconstructed from a large cohort of CT scans using the segmentation and quality-control workflow described previously Bartol et al. (2025). Briefly, low-quality scans, including scans with thick slices or incomplete thoracic coverage, were excluded before downstream geometry processing. The reconstruction pipeline produced 542 candidate lower-airway 3D reconstructed geometries as mesh surfaces. Automated geometric checks for non-physical branches, unrealistic dimensions, and related failures reduced this set to 412 anatomically valid lower-airway domains for population-level analysis via a downselection process explained below.

Each airway geometry i was summarized by a feature vector $z_{i}$,

(2) $$z_{i}={\left[t_{d,i},\;b_{a,i},\;V_{i},\;L_{i},\ldots \right]}^{⊤},$$

where $t_{d}$ is the volume-averaged tracheal diameter, $b_{a}$ is the main-bronchi carina angle, V is the segmented airway volume, and L is the cumulative bronchial length. Feature vectors were standardized as

(3) $${\tilde{z}}_{i}=D^{-1}\left(z_{i}-\mu \right),$$

with μ the cohort mean and D the diagonal matrix of feature standard deviations.

A Random Forest analysis in the prior downselection workflow identified $t_{d}$ and $b_{a}$ as informative descriptors for stratification. The reduced phenotype space was therefore defined as

(4) $$y_{i}={\left[{\tilde{t}}_{d,i},\;{\tilde{b}}_{a,i}\right]}^{⊤}.$$

Phenotypic groups were then obtained using the clustering and representative-selection procedure explained in Bartol et al. (2024b). In the present manuscript, this process yielded nine male groups and nine female groups, containing the 412 airway geometries. One representative geometry was selected from each group by choosing the nearest-neighbor exemplar to the reported cluster centroid,

(5) $$i_{k}^{⋆}=\text{arg}\;\underset{i\in {𝒞}_{k}}{\text{min}}\;{\left\|y_{i}-\mu_{k}\right\|}_{2},\;{𝒢}_{k}^{⋆}\equiv {𝒢}_{i_{k}^{⋆}}.$$

resulting in a total of 18 representative geometries for the database.

As a summary, the downselection progression was therefore starting with 542 candidate reconstructions, 412 anatomically valid lower-airway domains, 18 representative geometries after sex-stratified downselection with available particle-track simulations. Only nine male geometries entered the present generative-model study. Consequently, all trajectory-generation evidence in this manuscript concerns the nine male simulated geometries rather than the full cohort of 412 anatomically valid geometries. The reason for not including the female geometries in this study, is that derived particle tracks from the female cohort is still under validation studies and were not yet published.

#### 2.2.2. Carrier-phase airflow model

The reference carrier-phase airflow calculations used incompressible Reynolds-averaged Navier-Stokes (RANS) equations with the k-ω shear-stress-transport (SST) model Menter (1994) and the Langtry-Menter correlation-based transition model (SST-$\gamma -Re_{\theta }$, or SST-LM) Menter et al. (2006b); Langtry et al. (2004); Menter et al. (2006a). For a complete numerical please refer to the prior simulation work Bartol et al. (2025). The incompressible RANS equations were written as

(6) $$\nabla \cdot \bar{u}=0,$$

(7) $$\rho \left(\frac{\partial \bar{u}}{\partial t}+\bar{u}\cdot \nabla \bar{u}\right)\;=-\nabla \overset{‾}{p}+\nabla \cdot \left[\left(\mu +\mu_{t}\right)\left(\nabla \bar{u}+\nabla {\bar{u}}^{⊤}\right)\right]+S_{p},$$

where $\bar{u}$ is the mean velocity, $\overset{‾}{p}$ is the mean pressure, μ is the molecular viscosity, $\mu_{t}$ is the modeled eddy viscosity from the SST-LM closure, and $S_{p}$ is the particle back-coupling momentum source term described in Section 2.2.3.

A sinusoidal inlet-flow waveform representative of heavy exercise was prescribed at the tracheal inlet,

(8) $$Q\left(t\right)=Q_{\text{max}}\;\text{sin}\left(\frac{2\pi t}{T}\right),\;Q_{\text{max}}=100\;\text{L}{\;\text{min}}^{-1},\;T=2\;\text{s}.$$

The inlet bulk velocity magnitude was then set from the instantaneous flow rate and inlet area A,

(9) $$\left\|{\bar{u}}_{\text{in}}(t)\right\|=\frac{Q(t)}{A}.$$

Turbulence inlet quantities were specified from turbulence intensity I and a characteristic length scale L,

(10) $$k_{\text{in}}=\frac{3}{2}{\left(I\left\|{\bar{u}}_{\text{in}}\right\|\right)}^{2},\;\omega_{\text{in}}=\frac{k_{\text{in}}^{1/2}}{C_{\mu }^{1/4}L}.$$

We set the initial turbulence intensity at I=4%, and L defined as the geometry inlet hydraulic diameter $d_{h}$. A value of 0.09 was set for the $C_{\mu }$-related initialization constant.

#### 2.2.3. Lagrangian particle tracking and two-way coupling

Particles were simulated using a Lagrangian frame with two-way coupling between the particles and carrier phase. For particle p with mass $m_{p}$, position $x_{p}$, and velocity $v_{p}$, the particle equation of motion was formulated as

(11) $$\frac{\text{d}x_{p}}{dt}=v_{p},\;m_{p}\frac{\text{d}v_{p}}{dt}=F_{p}^{\left(\text{drag}\right)}+F_{p}^{\left(\text{grav}/\text{buoy}\right)}+F_{p}^{\left(\text{lift}\right)}+F_{p}^{\left(\text{vm}\right)}+F_{p}^{\left(\text{Brown}\right)}.$$

The modeled forces included drag, gravity/buoyancy, shear lift, virtual mass, and Brownian forces in the submicron regime. The closures and coefficients follow the prior CFPD work Bartol et al. (2025).

Two-way coupling was represented by adding the reaction force from particles to the Eulerian momentum equation as a volumetric source over each control volume $\Omega_{c}$,

(12) $$S_{p}\left(\Omega_{c}\right)=-\frac{1}{\left|\Omega_{c}\right|}\sum_{p\in \Omega_{c}}F_{p\to f},$$

where $F_{p\to f}$ denotes the force exerted by a particle on the fluid, which is equal and opposite to the force applied to the particle.

Particle size distribution followed a log-normal sampling parameterized by an activity median aerodynamic diameter and a geometric standard deviation, assuming spherical particles. For diameter d,

(13) $$p(d)=\frac{1}{d\;\text{ln}\left(\sigma_{g}\right)\sqrt{2\pi }}\text{exp}\;\left(-\frac{{\left[\text{ln}\left(d/d_{g}\right)\right]}^{2}}{2\;{\text{ln}}^{2}\left(\sigma_{g}\right)}\right).$$

The values used for this work were $d_{g}=0.42\;\mu \text{m}$ and $\sigma_{g}=3.5$.

Simulated particles were advanced in time until wall deposition, outlet escape, or the end of the simulation. Each exported reference track contained an event state in {in-flight, deposited, escaped}. In the DDPM implementation, however, it was learned only to the three spatial position coordinates. Deposited and escaped reference trajectories were therefore represented in the training tensor through their terminal positions and the last-value-hold operation described in Section 2.3.1.

For each representative geometry ${𝒢}_{k}^{⋆}$, simulations were run under the fixed waveform described above, and particle tracks were exported as time-ordered sequences

(14) $${𝒯}^{\left(r\right)}={\left\{\left(t_{\ell },x_{\ell },v_{\ell },d_{p},{\text{state}}_{\ell }\right)\right\}}_{\ell =1}^{L_{r}},$$

where r indexes an exported trajectory and $L_{r}$ varies with the termination or export time. The variables $v_{\ell },\;d_{p}$, and ${\text{state}}_{\ell }$ were retained in the reference export but were not part of the reported C=3 diffusion-model output.

### 2.3. Trajectory representation and geometric preprocessing

#### 2.3.1. Trajectory tensorization, time discretization, and variable-length handling

For training of the DDPM purposes, each exported trajectory ${𝒯}^{\left(r\right)}$ was resampled to a fixed length of K=1024 physical-time indices,

(15) $$t_{k}=k\Delta t,\;k=0,\ldots ,K-1,\;K=1024,\;\Delta t=0.001\;25\;\text{s}.$$

The learning state at time index k was the three-dimensional position,

(16) $$s_{k}\in R^{3},\;x={\left\{s_{k}\right\}}_{k=0}^{K-1}\in R^{K\times 3}.$$

The K×3 notation was used for the mathematical description of particle trajectories. For one-dimensional convolutional implementation, batches were arranged in channel-first form (B,3,K). The final physical-time index is (K-1)Δt=1.27875s, which for practical purposes is described throughout the manuscript as an approximately 1.28 s horizon. The justification to cut the simulation time from t=2s to t=1.28s is to avoid long-tail events since most of the particles were either deposited on the airway wall or escaped through an outlet at the selected cutoff time. Particle step size was selected relative to local cell size of approximately 1 mm and local particle velocity.

If a particle deposited on the airway wall or escaped through an outlet before $t_{K-1}$, the final valid position was extended by last-value hold,

(17) $$x_{k}=x_{k^{⋆}}\;\forall k\geq k^{⋆},$$

where $k^{⋆}$ is the last time index for which the trajectory position was updated. The approximately 1.28 s learning horizon was selected using an empirical histogram of last-updated times to reduce the amount of stationary terminal padding relative to the full 2 s waveform interval. Last-value hold creates zero displacement increments after termination and therefore contributed to near-zero finite-difference velocities and accelerations that later created an unrealistic representation in pooled kinematic distributions.

#### 2.3.2. Coordinate frames and normalization

Each airway geometry was associated with voxelization metadata: an origin $o\in R^{3}$, voxel spacing $h\in R^{3}$, and grid dimensions $d\in N^{3}$. World coordinates were mapped elementwise to voxel coordinates by

(18) $$x^{\left(\text{vox}\right)}=\frac{x^{\left(\text{world}\right)}-o}{h},$$

and then normalized to the coordinate range used by the SDF query,

(19) $$x^{\left(\text{norm}\right)}=2\left(\frac{x^{\left(\text{vox}\right)}}{d-1}\right)-1.$$

The same normalized coordinate system was used for the position channels and for trilinear SDF sampling during guided diffusion. De-normalization to physical coordinates was applied for trajectory analysis.

#### 2.3.3. Geometry voxelization and SDF construction

Each three-dimensional airway surface mesh was provided as an STL surface and voxelized to a binary lumen occupancy grid $B\in \{0,1{\}}^{D\times H\times W}$. The longest bounding-box dimension was fitted to a target maximum grid size, and zero-padding was applied if needed to produce the model input dimensions of 64 × 64 × 64. The resulting 64^3^ grid is an approximate representation of the detailed airway surface; it should not be interpreted as preserving every distal geometric feature of the original mesh. The binary occupancy grid was used for learned geometry conditioning.

A signed distance field $S\in R^{64\times 64\times 64}$ was computed from the voxelized representation and stored with the corresponding metadata (**o**, **h**, **d**). The SDF was used only at inference time as a soft geometric guidance term. Throughout the manuscript, the adopted convention is S(x)<0 inside the represented airway lumen, S(x)>0 outside the represented airway lumen Continuous SDF values were obtained by trilinear interpolation in the normalized coordinate frame used by Eq. (19).

**Figure 1:**
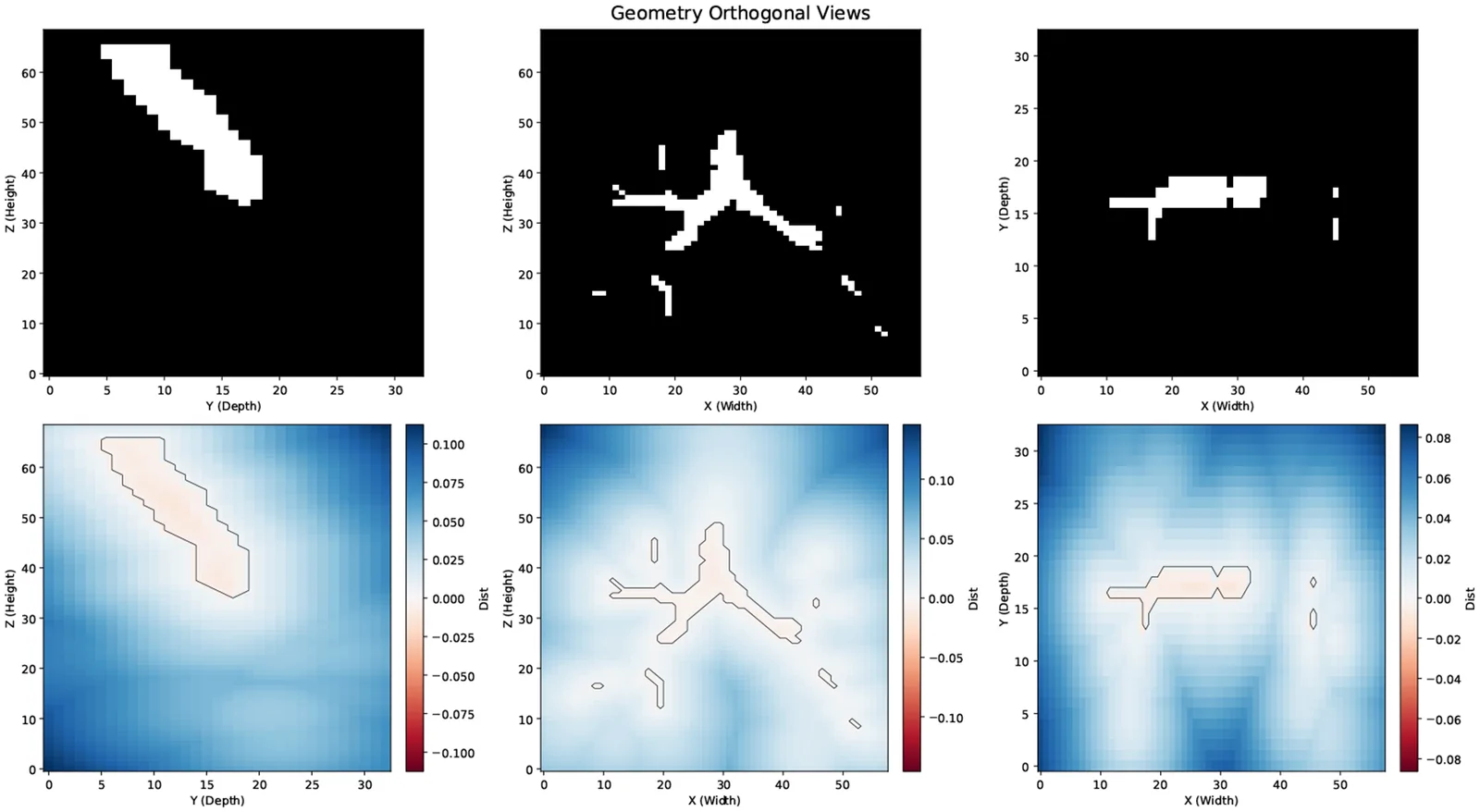
Binary occupancy and signed distance field for one airway geometry. Top: slices of the binary voxelized lumen supplied to the variational geometry encoder. Bottom: slices of the signed distance field used during inference-time guidance. The adopted sign convention in this manuscript is negative inside the represented lumen and positive outside. Voxelization provides an approximate representation of the STL airway surface.

The two geometry representations therefore had distinct roles: B provided the occupancy-based conditioning input to the denoiser, and S provided an inference-time geometric penalty. Figure 1 illustrates the binary grid and corresponding SDF for one geometry.

### 2.4. Geometry-conditioned diffusion model

Let $x\in R^{K\times 3}$ denote a fixed-length three-dimensional particle-position track, and let 𝒢 denote the airway geometrical field supplied through the occupancy grid. The model approximates a conditional distribution over position sequences, $p_{\theta }(x|𝒢)$, under the fixed inlet airflow waveform, particle size distribution, injection protocol, and preprocessing represented in the training data. The modeled object by the DDPM is the position trajectory over time. The training objective contains a denoising loss and a geometry-embedding KL regularization term.

Following diffusion probabilistic modeling Sohl-Dickstein et al. (2015); Ho et al. (2020); Song et al. (2021) and guided-diffusion implementations used in related trajectory-generation work Li et al. (2024a); Dhariwal and Nichol (2021), a fixed Markov chain gradually corrupts a clean trajectory $x_{0}$ obtained from the CFPD simulation with Gaussian noise, the physical time index is denoted by k, whereas the diffusion step index is denoted by n. We can write the well known forward process as

(20) $$q\left(x_{1:N}|x_{0}\right)=\prod_{n=1}^{N}\;q\left(x_{n}|x_{n-1}\right),\;q\left(x_{n}|x_{n-1}\right)=𝒩\left(\sqrt{\alpha_{n}}x_{n-1},\left(1-\alpha_{n}\right)I\right).$$

Each noise corruption step retains a fraction $\sqrt{\alpha_{n}}$ of the previous signal and adds independent Gaussian noise with variance $\beta_{n}=1-\alpha_{n}$. The forward process is independent of the airway geometry; geometry enters through the learned reverse process and the inference-time guidance described below in this section.

While the derivation of the closed-form marginal is derived in the original work by Sohl-Dickstein et al. (2015), for any diffusion step can be written as

(21) $$q\left(x_{n}|x_{0}\right)=𝒩\left(\sqrt{{\overset{‾}{\alpha }}_{n}}x_{0},\left(1-{\overset{‾}{\alpha }}_{n}\right)I\right),\;\Rightarrow \;x_{n}=\sqrt{{\overset{‾}{\alpha }}_{n}}x_{0}+\sqrt{1-{\overset{‾}{\alpha }}_{n}}\epsilon ,\;\epsilon \sim 𝒩(0,I).$$

This closed-form allows training examples to be generated by sampling n directly rather than simulating every intermediate noising step, which would be much more inneficient.cf

The implementation used in this work was N=800 diffusion steps and adopted the hyperbolic-tangent cumulative schedule tanh6,1 used in Li et al. (2024a),

(22) $${\overset{‾}{\alpha }}_{n}=\frac{-\text{tanh}\left(7\frac{n}{N}-6\right)+\text{tanh}(1)}{-\text{tanh}(-6)+\text{tanh}(1)},\;n\in \{0,\ldots ,N\},$$

which satisfies ${\overset{‾}{\alpha }}_{0}=1$ and ${\overset{‾}{\alpha }}_{N}≃0$. The per-step coefficients were then obtained as

(23) $$\alpha_{n}=\frac{{\overset{‾}{\alpha }}_{n}}{{\overset{‾}{\alpha }}_{n-1}},\;\beta_{n}=1-\alpha_{n},\;n=1,\ldots ,N.$$

For the reverse denoising process, the generative model learns a reverse-time Markov chain that approximately inverts the forward corruption:

(24) $$p_{\theta }\left(x_{0:N-1}|x_{N},𝒢\right)=\prod_{n=N}^{1}\;p_{\theta }\left(x_{n-1}|x_{n},𝒢\right).$$

The idea of conditioning the neural network on 𝒢 is to make the reverse process dependent on the anatomy, meaning that for a given random Gaussian noise initialization $x_{N}$, the denoising process is now conditioned on the encoded airway representation so now $p_{\theta }\left(x_{0:N-1}|x_{N}\to p_{\theta }\left(x_{0:N-1}|x_{N},𝒢\right)\right.$. Each reverse transition was modeled as a Gaussian distribution,

(25) $$p_{\theta }\left(x_{n-1}|x_{n},𝒢\right)=𝒩\left(\mu_{\theta }\left(x_{n},n,𝒢\right),\;\sigma_{n}^{2}I\right).$$

The reverse variance was fixed rather than learned as in previous literature, where $\sigma_{n}^{2}$ is set from the forward schedule, and $\sigma_{n}^{2}=\beta_{n}$ for most steps and the forward-posterior variance at the first reverse step.

The denoiser predicts the Gaussian noise added to a position-sequence tensor,

(26) $${\overset{ˆ}{\epsilon }}_{\theta }=\epsilon_{\theta }\left(x_{n},n,𝒢\right),$$

where $\epsilon_{\theta }$ is a one-dimensional temporal U-Net with the embedded conditioning. Given ${\overset{ˆ}{\epsilon }}_{\theta }$, the new position estimate is

(27) $${\overset{ˆ}{x}}_{0}\left(x_{n},n,𝒢\right)=\frac{1}{\sqrt{{\overset{‾}{\alpha }}_{n}}}\left(x_{n}-\sqrt{1-{\overset{‾}{\alpha }}_{n}}{\overset{ˆ}{\epsilon }}_{\theta }\right).$$

The reverse mean is then computed from the DDPM posterior parameterization Ho et al. (2020); Dhariwal and Nichol (2021),

(28) $$\mu_{\theta }\left(x_{n},n,𝒢\right)=\tilde{\mu }\left(x_{n},{\overset{ˆ}{x}}_{0}\right)=\frac{1}{\sqrt{\alpha_{n}}}\left(x_{n}-\frac{\beta_{n}}{\sqrt{1-{\overset{‾}{\alpha }}_{n}}}{\overset{ˆ}{\epsilon }}_{\theta }\right).$$

For numerical stability, the reconstructed ${\overset{ˆ}{x}}_{0}$ was clipped to the normalized coordinate range [−1, 1] to constrain reconstructed normalized positions to the training range.

### Training objective..

Training sampled a clean trajectory $x_{0}$, a diffusion step n, and Gaussian noise ϵ, then constructed $x_{n}$ using Eq. (21). The denoiser was trained with the standard noise-prediction objective loss function,

(29) $${ℒ}_{\text{ddpm}}(\theta )=E_{x_{0},n,\epsilon }\left[{\left\|\epsilon -\epsilon_{\theta }\left(x_{n},n,𝒢\right)\right\|}_{2}^{2}\right],\;n\sim \text{Uniform}\{1,\ldots ,N\},\;\epsilon \sim 𝒩(0,I).$$

Minimizing Eq. (29) trains the U-net $\epsilon_{\theta }$ to estimate the sampled noise at diffusion step n, conditioned on the encoded airway geometry. After training, new trajectories were generated by initializing $x_{N}\sim 𝒩(0,I)$ and iterating over the reverse process from n=N to 1 applying the SDF guidance described in Section 2.5.

Regarding the geometry conditioning, the diffusion model must generate trajectories in bounded anatomy-dependent domains. The occupancy grid therefore supplies a learned global representation of airway morphology to the temporal denoiser. This learned geometry conditioning is distinct from the SDF guidance term, the occupancy grid is encoded by the neural network, whereas the SDF is queried directly during inference as a soft penalty.

#### 2.4.1. Global geometry encoder and variational bottleneck

Given a voxelized occupancy grid $B\in \{0,1{\}}^{1\times D\times H\times W}$, a three-dimensional variational geometry encoder maps the occupancy volume to a global stochastic embedding. In compact notation,

(30) $$z_{𝒢}=f_{\phi }(B),\;z_{𝒢}\in R^{d_{e}}.$$

The purpose of $f_{\phi }$ is to compress the high-dimensional occupancy volume into a low-dimensional vector supplied to the denoiser. The study does not directly evaluate how much local or distal-branch information is retained in this global representation.

The encoder outputs distribution parameters,

(31) $$\left(\mu_{𝒢},\text{log}\;\sigma_{𝒢}^{2}\right)=f_{\phi }(B),\;\mu_{𝒢},\sigma_{𝒢}\in R^{d_{e}},$$

and samples the embedding through reparameterization,

(32) $$z_{𝒢}=\mu_{𝒢}+\sigma_{𝒢}⊙\eta ,\;\eta \sim 𝒩(0,I),$$

where ⊙ denotes elementwise multiplication. The Kullback–Leibler (KL) regularization term was

(33) $${ℒ}_{\text{KL}}=D_{\text{KL}}\left(𝒩\left(\mu_{𝒢},\sigma_{𝒢}^{2}\right)\|𝒩(0,I)\right).$$

The complete training objective for the conditioned DDPM was

(34) $${ℒ}_{\text{total}}={ℒ}_{\text{ddpm}}+\lambda_{\text{KL}}{ℒ}_{\text{KL}},\;\lambda_{\text{KL}}=10^{-4}.$$

The KL term pulls the geometry-embedding distribution toward the prior and was intended to discourage the encoder from memorizing the discrete training geometries as isolated codes. It does not, on its own, establish that anatomically similar airways map to nearby latent points or that the latent space is organized in an interpretable way. Because the module has no decoder and no geometry-reconstruction objective, we refer to it as a variational geometry encoder rather than as a decoder-based reconstruction model.

The trajectory denoiser $\epsilon_{\theta }\left(x_{n},n,𝒢\right)$ receives the embedded geometry through the same embedding pathway used for diffusion-step conditioning. The reported operation adds the geometry embedding to the timestep embedding,

(35) $$e_{\text{cond}}(n,𝒢)=e(n)+z_{𝒢}.$$

If the timestep and geometry embeddings are not already dimension matched, a learned projection was done before the sum in (35). The resulting $e_{\text{cond}}$ conditions each residual block in the temporal U-Net on both the diffusion step and the encoded airway representation.

Following the guided-diffusion U-Net style Nichol and Dhariwal (2021); Dhariwal and Nichol (2021), each residual block receives an embedding vector. With $h_{\ell }$ denoting the feature map at block ℓ, the conditioned residualblock schema is

(36) $$h_{\ell +1}=h_{\ell }+\text{Res}\left(h_{\ell },e_{\text{cond}}(n,𝒢)\right).$$

and the evaluated implementation injects the embedding as a projected additive bias inside the residual transformation,

(37) $$\text{Res}\left(h_{\ell },e_{\text{cond}}\right)={\text{Conv}}_{2}\left(\sigma \left(\text{GN}\left({\text{Conv}}_{1}\left(\sigma \left(\text{GN}\left(h_{\ell }\right)\right)\right)+W_{\ell }e_{\text{cond}}\right)\right)\right),$$

where σ(⋅) is the SiLU activation, GN is group normalization, and $W_{\ell }$ is a learned projection. This is an additive-bias mechanism, not FiLM scale-and-shift modulation Perez et al. (2018); which we describe in Eq. (37).

### 2.5. SDF-guided diffusion sampling

Occupancy conditioning does not guarantee that every stochastic sample remains within the represented lumen. During inference, an SDF-derived formulation, which can be interpreted as a repulsive energy, was therefore used to bias reverse diffusion away from out-of-lumen positions. This guidance is a soft geometric penalty, not a physical wall-contact model and not a hard containment guarantee, specially since the DDPM is a denoising process, which does not generate particle tracks one step at a time. The SDF grid $S\in R^{D\times H\times W}$ shares the geometry grid with the occupancy representation, and S(x) denotes a continuous SDF value queried at a trajectory position.

To formulate the energy-like SDF penalty, let $x_{n}\in R^{K\times 3}$ denote the current noisy trajectory at diffusion step n, with $x_{n,k}\in R^{3}$ the position at physical-time index k. Using the negative-inside and positive-outside convention from Section 2.3.3, the out-of-lumen energy becomes

(38) $$E\left(x_{n};S\right)=\sum_{k=0}^{K-1}\;\psi \left(S\left(x_{n,k}\right)\right).$$

The hinge penalty was

(39) ψ(s)=ReLU(s).

Thus, generated trajectories that have points with S(x)<0 do not contribute to the hinge penalty, while points with S(x)>0 contribute in proportion to the interpolated positive SDF value. Away from the non-differentiable boundary of the hinge penalty, the contribution of a single point is

(40) $$\nabla_{x_{n,k}}E\left(x_{n};S\right)=\left\{\begin{array}{ll} \nabla_{x}S\left(x_{n,k}\right), & S\left(x_{n,k}\right)>0,\\ 0, & S\left(x_{n,k}\right)<0. \end{array}\right.$$

The descent direction $-\nabla_{x_{n,k}}E$ is intended to move positive-SDF points toward lower SDF values and the represented lumen. Because the SDF is interpolated on an approximate voxel grid, this operation is as an inference-time bias rather than a guarantee that all samples remain in-lumen.

#### 2.5.1. Guidance-modified reverse step

In standard DDPM sampling, one reverse step is

(41) $$p_{\theta }\left(x_{n-1}|x_{n},𝒢\right)=𝒩\left(\mu_{\theta }\left(x_{n},n;𝒢\right),\;{𝚺}_{n}\right),$$

where $\mu_{\theta }$ is computed from the denoiser as described in equation 28. The reported guided sampler shifted the reverse mean using the SDF energy descent direction,

(42) $$\tilde{\mu }\left(x_{n},n;𝒢,S\right)=\mu_{\theta }\left(x_{n},n;𝒢\right)-\lambda_{g}\nabla_{x_{n}}E\left(x_{n};S\right),$$

with guidance scale $\lambda_{g}>0$. The value of $\lambda_{g}=1.0$ was set as the default value, and in the experiments ranged from 1.0 to 4.0, adjusted on the fly to reduce the amount of trajectories that drifted away from the airway lumen. The guided reverse sample was then

(43) $$x_{n-1}=\tilde{\mu }\left(x_{n},n;𝒢,S\right)+{𝚺}_{n}^{1/2}z,\;z\sim 𝒩(0,I).$$

This mean shift is described as the implemented SDF-guided sampling rule. It should not be read as a claim that the method implements covariancescaled classifier Dhariwal and Nichol (2021).

#### 2.5.2. Sampling algorithm

Algorithm 1 summarizes the geometry-conditioned sampling procedure. The occupancy grid B conditions the denoiser throughout the reverse process, while the SDF S contributes through the gradient term in Eq. (42). Physicalworld coordinates of the trajectories are obtained by de-normalizing $x_{0}$ after sampling.

### 2.6. Network architecture and training protocol

The last part related to the DDPM is the actual model architechture. The denoiser was a one-dimensional U-Net operating along the trajectory-time dimension. Its input shape was (B,C,K), with C=3 position channels and K=1024 time indices, and its output was $\overset{ˆ}{\epsilon }\in R^{B\times C\times K}$. The U-Net followed the guided-diffusion design Nichol and Dhariwal (2021); Dhariwal and Nichol (2021), with base channel count 128, channel multipliers (1, 1, 2, 3, 4), and 4 attention heads.

Training used AdamW with an initial learning rate of 10^−4^, batch size 128 or 64 depending on the GPU configuration, and an exponential moving average (EMA) of model weights. Multi-GPU training used MPI/DDP following the implementation pattern in Li et al. (2024a). The available hardware was either one NVIDIA H100 GPU or two NVIDIA A100 GPUs, each with 80 GB of memory. The GitHub repository provides all the implementation and configuration details.

**Algorithm 1:** Geometry-conditioned diffusion sampling with SDF-based soft guidance

**Require:** Occupancy grid B representing 𝒢, SDF S, metadata (**o**, **h**, **d**), guidance scale $\lambda_{g}$, diffusion steps N	
1:	Initialize $x_{N}\sim 𝒩(0,I)$ in normalized coordinate space
2:	**for** n=N,N-1,…,1 **do**
3:	Predict noise: $\overset{ˆ}{\epsilon }\leftarrow \epsilon_{\theta }\left(x_{n},n;B\right)$
4:	Compute DDPM mean: $\mu_{\theta }\left(x_{n},n;B\right)$ from $\overset{ˆ}{\epsilon }$
5:	Evaluate SDF energy: $E\leftarrow \sum_{k}\;\text{ReLU}\left(S\left(x_{n,k}\right)\right)$
6:	Compute soft-guidance direction: $g\leftarrow -\nabla_{x_{n}}E$
7:	Shift mean: $\tilde{\mu }\leftarrow \mu_{\theta }+\lambda_{g}g$
8:	Sample: $x_{n-1}\leftarrow \tilde{\mu }+{𝚺}_{n}^{1/2}z,z\sim 𝒩(0,I)$
9:	**end for**
10:	De-normalize $x_{0}$ to world coordinates when reporting trajectories in physical units
11:	**return** ${\text{x}}_{0}$

**Figure 2:**
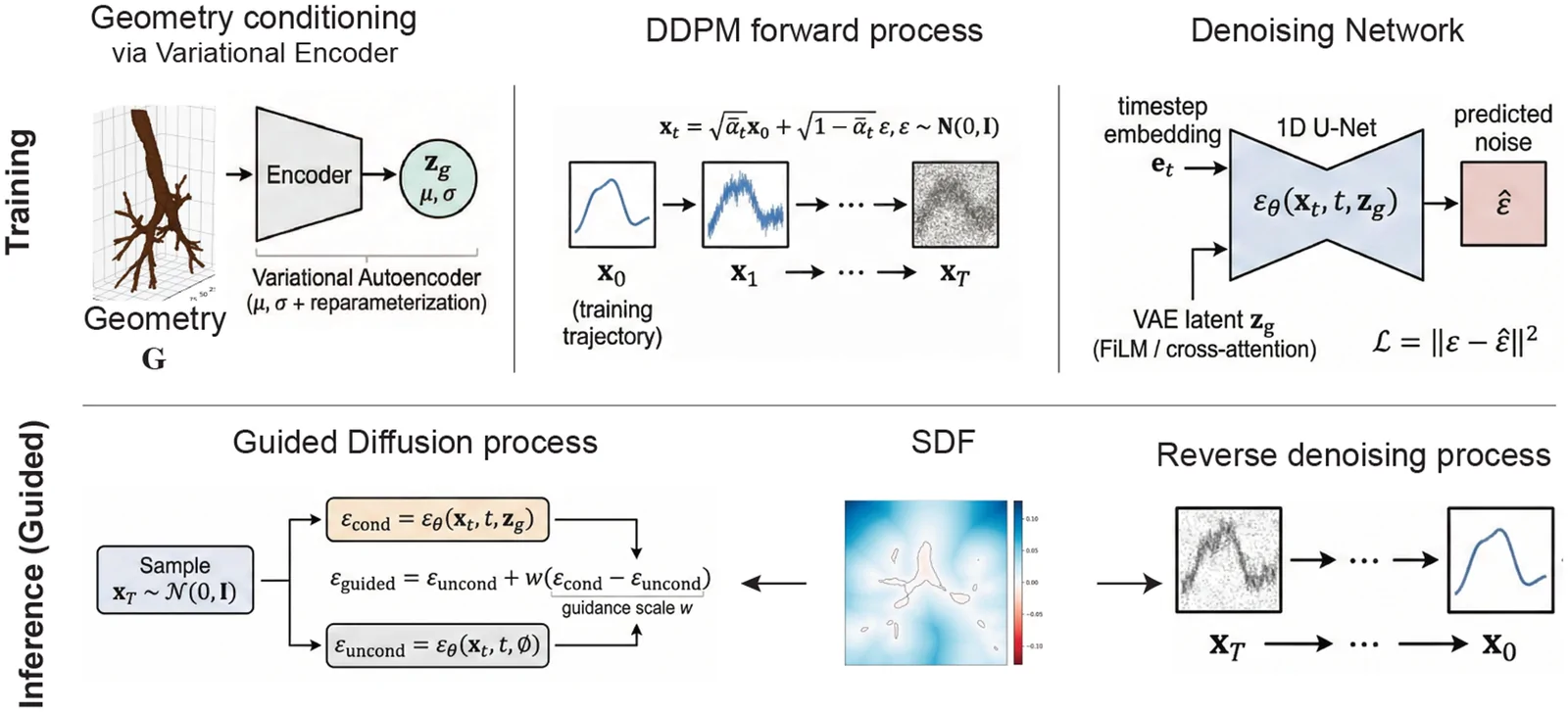
Methodological overview of the geometry-conditioned guided DDPM. The top row summarizes training. A subject-specific airway geometry is voxelized and encoded with a variational geometry encoder to obtain a stochastic global anatomy embedding. A clean fixed-length particle-position trajectory is progressively corrupted by the forward diffusion process, and a one-dimensional U-Net denoiser is trained to predict the injected noise while conditioned on the diffusion step and encoded geometry. The bottom row summarizes inference. Starting from Gaussian noise, the reverse process generates a position trajectory. The occupancy grid provides learned anatomical conditioning, while the SDF supplies an inference-time soft guidance term intended to reduce out-of-lumen samples. The guidance term does not enforce an exact physical boundary or predict terminal deposition or escape states.

Figure 2 provides a graphical summary of the training and guided-inference workflows. The upper row presents the three principal components of training. First, the voxelized airway geometry is mapped to a stochastic anatomy embedding using a variational encoder. In parallel, a clean particle trajectory is progressively corrupted through the DDPM forward process according to a prescribed noise schedule, such that the trajectory distribution approaches a standard Gaussian distribution at the terminal diffusion step. The resulting noisy trajectory, diffusion-step embedding, and geometry latent are then provided to the one-dimensional U-Net, which is trained to predict the injected noise at randomly sampled diffusion steps. Model parameters are optimized by minimizing the discrepancy between the predicted and applied noise.

The lower row illustrates the guided inference procedure. Sampling begins from Gaussian noise, and the airway geometry is again encoded to condition the reverse denoising process. Conditional and unconditional noise estimates are combined through classifier-free guidance, while an additional SDF-based guidance term penalizes trajectory points predicted outside the airway lumen. This soft geometric constraint biases the reverse process toward anatomically feasible particle tracks, although it does not impose an exact physical boundary condition or explicitly model particle-wall interactions.

### 2.7. Evaluation design and trajectory-derived metrics

Generated trajectories were compared with the CFPD simulation-derived reference trajectories using the existing qualitative and quantitative summaries which will be described in detail in this section. The qualitative comparisons use projected and three-dimensional overlays of reference and generated tracks over the airway geometry. The quantitative comparisons use speed magnitude distributions, path-length and acceleration summaries, Wasserstein-1 distance for pooled speed distributions, and, a further analysis was used comparting DDPM to DMD reduced order model outputs. Table 1 reports the trajectory-derived kinematic summaries and speed-distribution distances for the main DDPM evaluation.

The definitions below use the position trajectory ${\left\{x_{k}\right\}}_{k=0}^{K-1}$ from Eq. (16). For both reference and generated tracks, finite-difference quantities were computed from positions on the fixed grid with time step Δt. Metrics pooled over all time indices excluded stationary terminal segments introduced by last-value hold. If included, these segments add zero or near-zero increments after a track terminates, affecting the interpretation of pooled speed and acceleration distributions.

#### 2.7.1. Kinematic metrics

The velocity was computed as forward finite-difference associated with the interval from k to k+1 as,

$${\hat{v}}_{k}=\frac{x_{k+1}-x_{k}}{\Delta t},\;k=0,\ldots ,K-2.$$

The speed magnitude was defined as

$$s_{k}={\left\|{\hat{v}}_{k}\right\|}_{2},$$

where $\|\cdot {\|}_{2}$ is the Euclidean norm. Speed has units of m s^−1^. The reported speed summaries include the mean E[s], standard deviation Std[s], and 99^th^ percentile p99[s].

Acceleration magnitude was approximated by a first-order finite difference of the velocities,

$$a_{k}=\frac{{\left\|{\hat{v}}_{k+1}-{\hat{v}}_{k}\right\|}_{2}}{\Delta t},\;k=0,\ldots ,K-3.$$

Acceleration is reported in units of m s^−2^. The reported acceleration summaries include the mean E[a] and 99^th^ percentile p99 [a].

Another metric analyzed was the path length for a trajectory; which wass computed from position increments,

(44) $$L=\sum_{k=0}^{K-2}\;{\left\|x_{k+1}-x_{k}\right\|}_{2}.$$

Path length has units of meters.

#### 2.7.2. Distributional similarity

The one-dimensional Wasserstein-1 distance was used to compare empirical pooled speed distributions for generated and reference trajectories Gabriel and Marco (2019). We define $F_{\text{gen}}$ and $F_{\text{ref}}$ to denote the empirical cumulative distribution functions of the generated and reference speeds. Then

(45) $$W_{1}\left(F_{\text{gen}},F_{\text{ref}}\right)=\int_{-\infty }^{\infty }\;\left|F_{\text{gen}}(u)-F_{\text{ref}}(u)\right|du.$$

For speed distributions, $W_{1}$ has units of m s^−1^. While there is no strict lower bound, lower values indicate closer pooled speed distributions under this metric.

### 2.8. Exploratory DMD baseline comparison

The DMD comparison from previous work by Graffigna et al. (2026) was retained as an exploratory baseline focused on particle-track representation. DMD is a deterministic, geometry-specific reconstruction fitted to a fixed ensemble state, whereas the DDPM developed in this work is a conditional stochastic generator of subject-specific fixed-length trajectory samples. DMD and multiresolution extensions are described in the cited literature SCHMID (2010); ROWLEY et al. (2009); Kutz et al. (2016); the present manuscript uses them only as a contrast for selected trajectory diagnostics. The comparison was restricted to a single geometry, namely Geometry 1, for which the DDPM was trained and the DMD operator was fitted.

The DMD reconstructed particle trajectories was splitted in two tensors, one with an all-particle tracks in a single tensor (both stuck and particles that have scaped) and a “stuck,” tensor, retaining only particles that were stuck to the lumen wall. Post hoc jump filtering for DMD was applied before computing derivative-sensitive diagnostics. A step exceeding 5×10^−2^ m was flagged. Trajectories with multiple flagged jumps were excluded, and trajectories with one flagged jump were truncated at the first flagged index. Tracks with fewer than 55 valid frames were excluded. The filtered comparison retained 404 all-tracks trajectories and 290 stuck trajectories, for 694 total retained DMD trajectories. The 404 retained all-tracks trajectories corresponded to 2.3% of the reported 17,840 all-tracks DMD output. This filtering reduced the influence of discontinuities on finite-difference velocities and spectra, but it also biased the DMD sample toward the most stable reconstructions.

The DMD exports contained 60 and 55 frames for the two groups. DDPM and reference trajectories were sampled on a common 55-frame grid using indices selected from the valid 0–1023 range of the 1024-element sequence. Under the adopted effective mapping, $\Delta t\approx 2.370\times 10^{-2}\;\text{s}$. Because the DMD and DDPM/reference tensors were produced through different time pipelines, lag and spectral diagnostics are best interpreted as frame-matched comparisons. When seconds or hertz are reported for this comparison, they refer to the adopted effective mapping.

The DMD diagnostics included scalar marginal comparisons, component-velocity distributions, multi-lag increment statistics, autocorrelation, and power spectral density. For scalar marginals, the reported analysis used Wasserstein-1 distance, the Kolmogorov-Smirnov statistic, and energy distance Ramdas et al. (2017); Massey (1951); Székely and Rizzo (2013). The corresponding filtered comparison is reported in Figure 7, Figure 11, Figure 12, and Table 2. Any reported comparison between DMD and guided DDPM should therefore be read as an exploratory, filtered, single geometry contrast rather than as a definitive ranking of the two modeling approaches.

## 3. Results

We evaluated the geometry-conditioned guided DDPM by comparing its generated three-dimensional particle-position trajectories against simulation-derived reference trajectories. Four questions organize the section: how SDF guidance affects apparent trajectory containment and airway-topology alignment; how closely generated tracks agree with the reference on training and held-out airway geometries; how well selected trajectory-derived kinematic distributions and summary metrics match; and how the guided DDPM compares, with a DMD particle-track reconstruction, which is another data-driven reduced order model. Deposited dose, regional deposition, escape probability, and categorical terminal-event prediction were not evaluated.

A note on sample counts for the figures, for visual clarity, the trajectory-overlay figures display 256 randomly selected trajectories out of the 16,384 generated for each set, whereas the distributional summaries use the full reported counts rather than the displayed subset. In the multi-geometry velocity analysis, the source reports 16,384 generated and 50,000 reference trajectories, and the DMD comparison later reports 16,384 trajectories of 1,024 timesteps, only for a single geometry.

### 3.1. Qualitative effect of SDF guidance on generated trajectories

**Figure 3:**
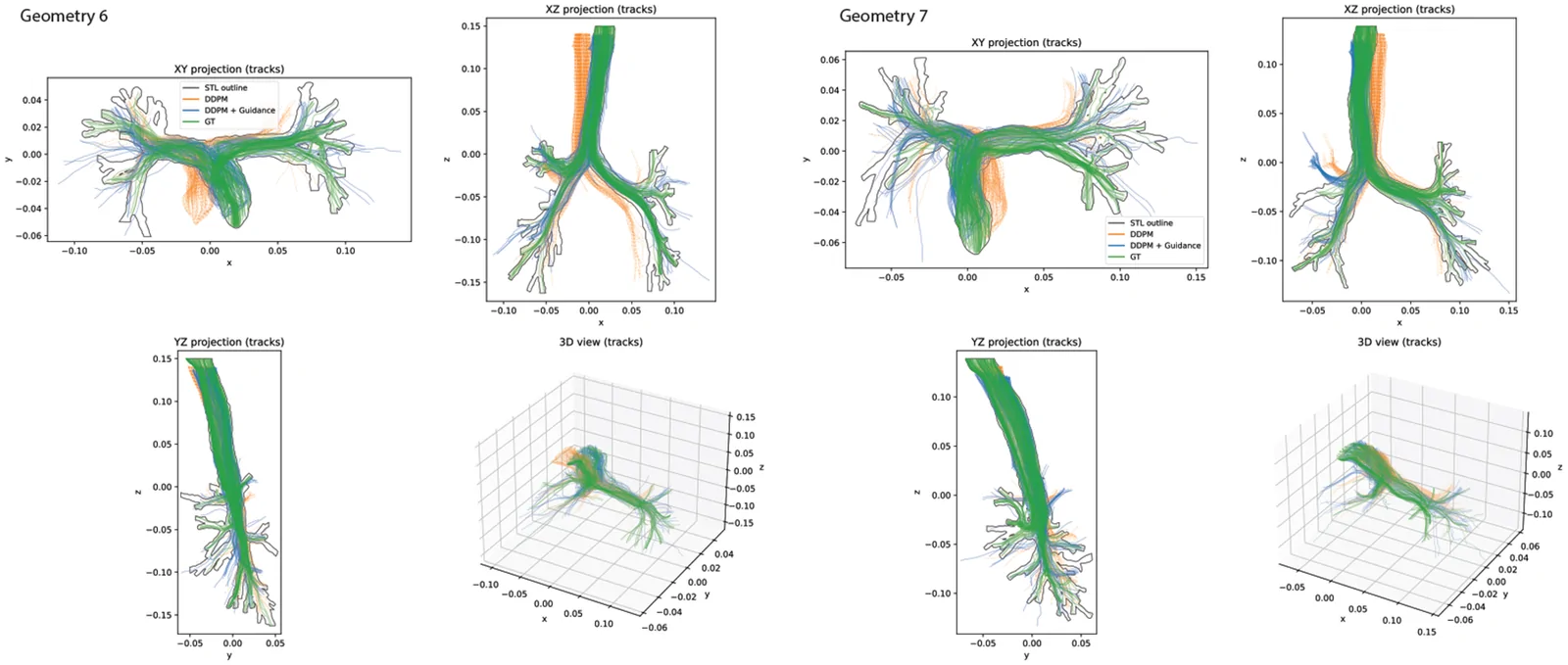
Qualitative effect of SDF guidance on generated particle trajectories. Reference trajectories (green), unguided DDPM samples (orange), and SDF-guided DDPM samples (blue) are shown for Geometries 6 and 7. Each geometry is displayed in the x-y,x-z, and y-z projections and in a three-dimensional view; the airway surface outline is shown in gray. Guided samples show fewer visually apparent out-of-lumen segments in these examples, but SDF acts as a soft bias and does not guarantee complete lumen containment.

Figure 3 shows reference trajectories, unguided DDPM samples, and SDF-guided DDPM samples for two reported airway geometries. In the unguided samples, some trajectory segments visibly extend outside the displayed airway outline, and several tracks do not follow the reference bifurcation pattern in the projected views. These visual discrepancies are clear in the tracheal and main bronchial regions, where the unguided samples drift laterally relative to the represented lumen.

On the other hand, the SDF-guided trajetctories display lower visually apparent out-of-lumen positions and closer alignment with the represented major branches. While this is an improvement, SDF guidance was used as a soft inference-time geometric bias; it does not model wall-interaction physics, therefore there are still some boundary inconsistencies.

### 3.2. Trajectory generation on training and held-out airway geometries

**Figure 4:**
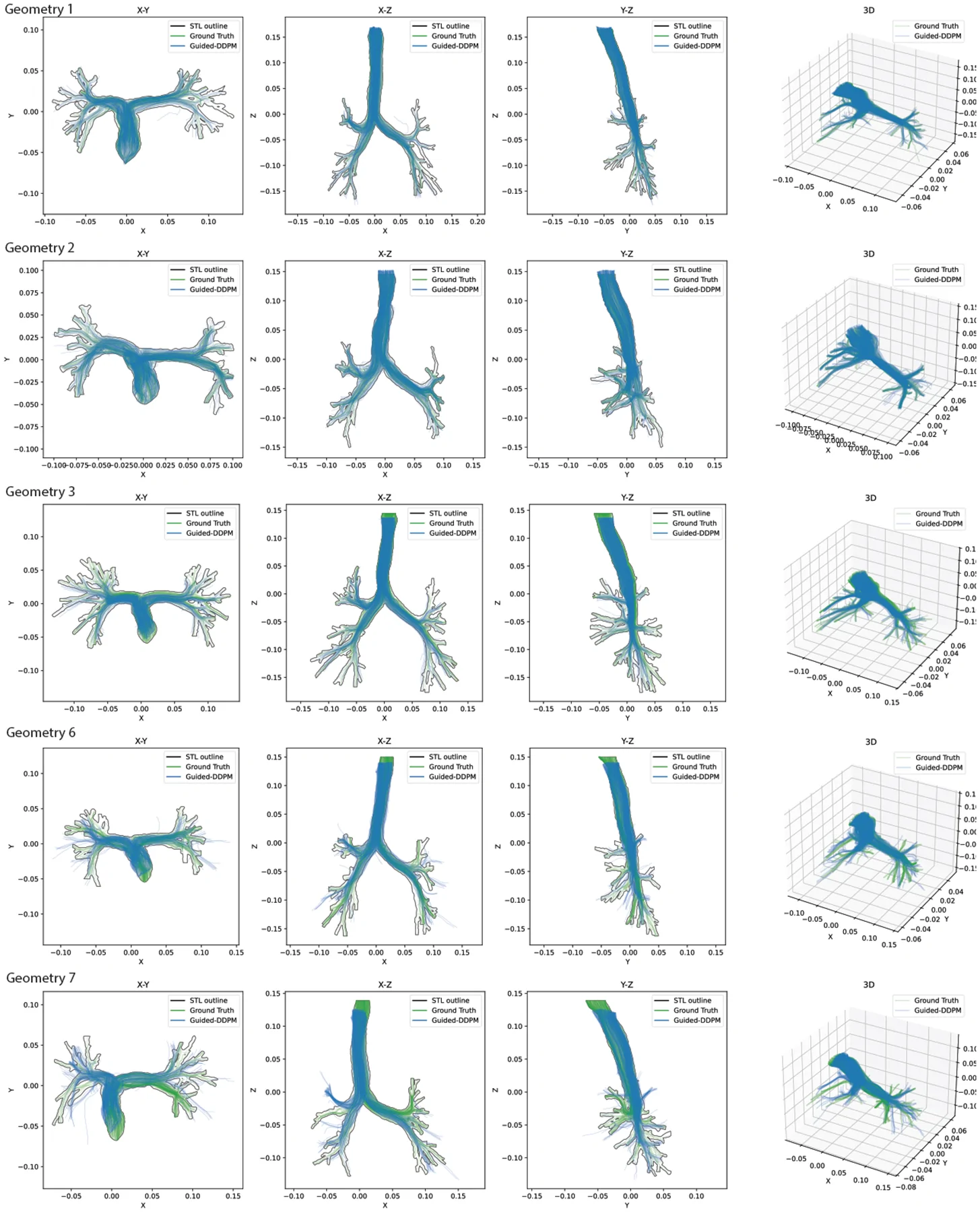
Reference and guided DDPM trajectories across reported airway geometries. Five airway geometries are shown with particle trajectories projected onto the x-y,x-z, and y-z planes and in a three-dimensional view. Reference trajectories are shown in green, guided DDPM samples are shown in blue, and the airway surface outline is shown in gray. Geometries 1 and 2 are reported as training geometries, whereas Geometries 3, 6, and 7 are reported as held out. The figure provides a qualitative comparison on the stated split and should not be read as proof of broad generalization.

Figure 4 summarizes qualitative trajectory agreement across five reported geometries. Geometries 1 and 2 are described as training geometries, whereas Geometries 3, 6, and 7 are described as held-out geometries. For the training geometries, the generated trajectories follow the displayed major airway pathways and branch-wise splitting patterns in the projections. Differences remain visible in smaller distal branches, where generated trajectories appear smoother or less spatially dispersed than the reference trajectories. For the reported held-out geometries, the geometry-conditioned sampling framework generated trajectories that are in agreement with the global airway topology, including the main airway and primary bifurcations. Discrepancies increased in distal branches, where generated tracks may under-represent peripheral pathways, exhibit lateral offsets relative to the surface outline, or concentrate in one dominant branch compared with the reference ensemble. These findings provide preliminary evidence of anatomy-conditioned trajectory generation on the stated held-out cases and establish potential for broader anatomical robustness.

Two sources of test-geometry information are present in the reported framework, first the voxelized occupancy grid is passed through the learned variational geometry encoder, which provides a global conditioning embedding. Separately, the SDF for the same geometry is supplied directly during reverse diffusion as an inference-time soft geometric guide. Therefore, heldout topology alignment and apparent containment are not solely attributed to the learned geometry embedding.

Due to the cost of CFPD simulations, only nine simulated airway geometries had reference particle trajectories used for training/testing the generativemodel. The qualitative held-out results should consequently be interpreted within the limited anatomy set. Additional simulation-derived trajectories from a more diverse anatomy set would be useful for future evaluation.

### 3.3. Velocity magnitude and trajectory-derived statistics

**Figure 5:**
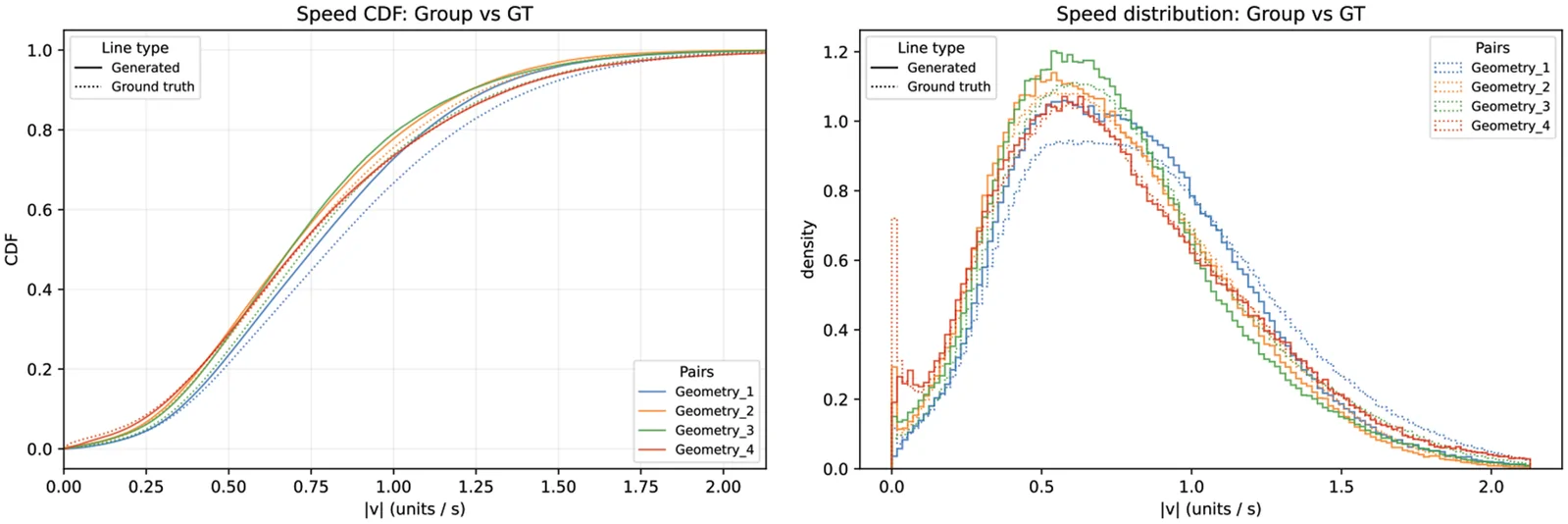
Velocity-magnitude distributions for Geometries 1–4. Cumulative distribution functions (left) and probability-density histograms (right) compare generated trajectories with reference trajectories. Velocity magnitudes were derived from position differences. Last-value-held terminal segments contribute near-zero mass, so the low-speed region should be interpreted together with the terminal-padding representation described in the Methods.

**Figure 6:**
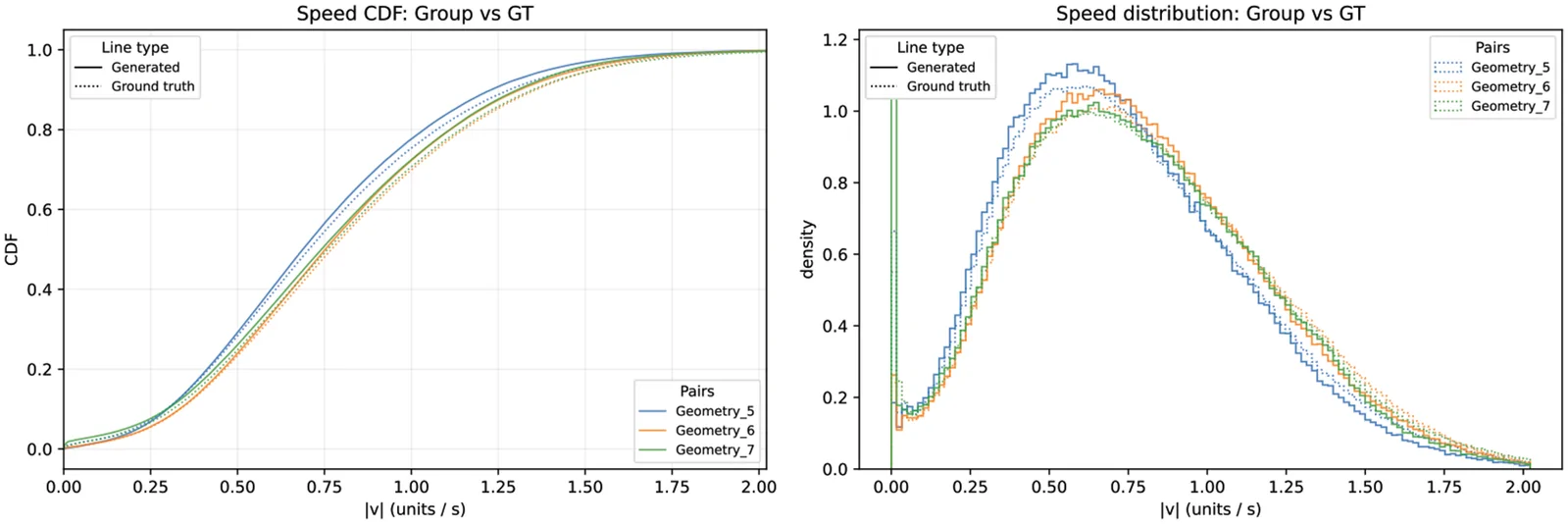
Velocity-magnitude distributions for Geometries 5–7. Cumulative distribution functions (left) and probability-density histograms (right) compare generated trajectories with reference trajectories. Residual discrepancies are visible near the stationary component and in the upper tail for some geometries. Velocity magnitudes were computed from position differences, and last-value-held terminal segments contribute to the near-zero component.

Velocity magnitudes were derived from first-order finite differences of generated and reference position trajectories. For the multi-geometry velocity comparison, the reported sample counts were N=16,384 generated trajectories and N=50,000 reference trajectories. Figures 5 and 6 show cumulative distribution functions and probability-density histograms for velocity magnitude across the analyzed geometries. Geometry 8 was excluded from this analysis because there was a large mismatch between the generated particle tracks by the DDPM and the represented geometry, despite agreement in part of the central velocity range.

Across the displayed geometries, the generated and reference cumulative distributions agree most closely in the central portion of the speed range. The histograms also show similar distributional shapes in several cases, while residual differences remain near the zero-velocity component and in the high-speed tails. The use of diffusion models for trajectory-sampling problems is consistent with the broader modeling context discussed by Li et al. (2024a), and the present evidence is limited to the reported airway trajectory validation.

Regarding quantitative analysis, percentage differences for the evaluation set with K=1024 (timesteps) and Δt=0.00125 were computed. The DDPM generated mean speed differed by approximately −4.3%, and the 99^th^ percentile speed differed by approximately −12.0%. Similar reported differences were given for mean path length (−4.3%), mean acceleration (−3.8%), and acceleration 99^th^ percentile (−11.5%).

Taken these results together, the qualitative trajectory overlays and velocity-distribution comparisons show that the guided DDPM generated position trajectories that followed the reported airway topology and reproduced trajectory kinematics to varying degrees. The evidence remains limited by the small number of simulated geometries, by the position-only trajectory representation, and by the absence of direct evaluation of terminal outcomes. The results should therefore be interpreted in the light of a proof-of-concept trajectory-generation evaluation.

### 3.4. Quantitative trajectory metrics

Table 1 reports acceleration, path-length, speed, and speed-distribution values for the analyzed HRT geometries. The metric definitions are provided in Section 2.7. The reported $W_{1}$ column compares pooled speed distributions between generated and reference trajectories, with units of m s^−1^. Lumen containment was over 95% for all geometries, and the reported metrics were computed on the retained trajectories including out-of-lumen segments.

The table includes Geometries 1, 2, 3, 4, 6, and 7. Based on the split stated in the trajectory-overlay results, Geometries 1, 2, and 3 were seen by the encoder during training and Geometries 4, 6, and 7 are held-out cases.

Across the reported geometries, mean values were generally closer than extreme percentiles, while the magnitude of the discrepancy varied by anatomy. The speed $W_{1}$ values also varied across cases, with lower values indicating closer pooled speed distributions relative to the reference for each of the geometroes.

### 3.5. Exploratory comparison with DMD for Geometry 1

#### 3.5.1. Evaluation setup

This comparison is an exploratory contrast between two data-driven approaches. The guided DDPM is a conditional stochastic generator of individual particle-position trajectories, using a learned geometry embedding and SDF guidance during sampling. The DMD reconstruction on the other hand is a deterministic, single-geometry reduced-order reconstruction fitted to a fixed particle-ensemble state. The results below should not be interpreted as a general benchmark of surrogate quality or as evidence that one approach replaces the other, but a comparison of their respective strengths and limitations.

The DMD particle-track reconstruction was taken from the DMD study Graffigna et al. (2026). In that formulation, the three Cartesian coordinates of all particles at a given time were shifted by injection time, $\tilde{t}=t-t_{\text{injected}}$, and stacked into a single state tensor. A reduced linear operator was then fitted to advance the ensemble state in time. The Koopman operator identifies an approximate linear evolution operator from paired snapshot matrices $X^{-}=\left[x_{1},\ldots ,x_{m-1}\right]$ and $X^{+}=\left[x_{2},\ldots ,x_{m}\right]$, so that $x_{k+1}\approx {Ax}_{k}$; the eigenvalues and modes of A define exponential space-time components SCHMID (2010); ROWLEY et al. (2009). Multiresolution DMD extends this idea by recursively applying DMD over time windows to separate dynamics across temporal scales Kutz et al. (2016). Here, DMD was used only as a particletrack reconstruction baseline for the reported Geometry 1 comparison.

The comparison was restricted to Geometry 1 because the DMD operator was fitted in one anatomical domain and cannot be applied unchanged to a new airway mesh without generating new snapshots, mapping states between geometries, and re-identifying the Koopman operator. The guided DDPM samples and the DMD reconstruction were therefore compared on the same geometry. The comparison focuses on particle-track representation, spatial continuity, and same kinematic distributions. The DMD reconstruction was fitted to two exported tensors: one constructed from particles stuck at the wall and another including all particles that either escaped the domain or terminated at the wall. These event categories describe the source trajectory exports and DMD inputs; the guided DDPM results reported here remain position-sequence outputs and do not include an explicit terminal-event classifier.

**Figure 7:**
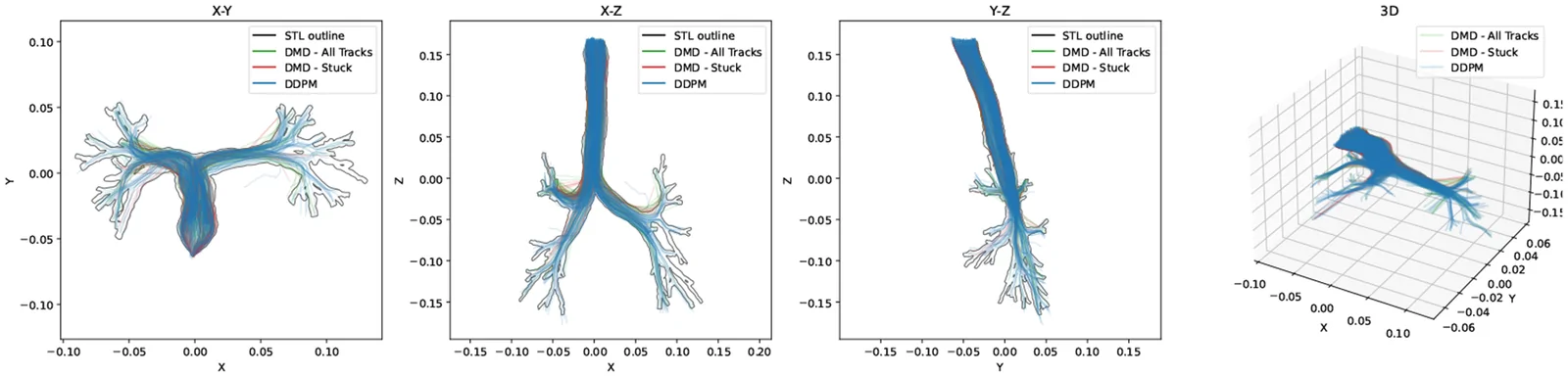
Exploratory Geometry 1 track comparison between guided DDPM and DMD. The black curves denote the STL outline, green curves denote the DMD all-tracks group, red curves denote the DMD stuck-particle group, and blue curves denote guided DDPM samples. Both outputs occupy major portions of the primary airway envelope, but the unfiltered DMD output includes isolated discontinuous return-to-origin segments. The derivative-based DMD diagnostics below use the jump-filtered subset described in the text and should be interpreted as a filtered Geometry 1 comparison.

Figure 7 gives the qualitative Geometry 1 comparison. The guided DDPM samples occupy the primary airway geometry in the X-Y,X-Z,Y-Z, and three-dimensional views. The DMD reconstruction also recovers major portions of the displayed branches, but some DMD trajectories contain abrupt non-physical discontinuous segments that return toward the coordinate origin near the carina. These discontinuities are reconstruction artifacts that come from the DMD fitting procedure, and they are not present in the reference trajectories. The DMD tracks artifacts were filtered out before derivative-based metrics were computed, as described below.

The DMD tracks were filtered before derivative-based metrics were computed. A particle track step was flagged when its magnitude exceeded 5 × 10^−2^ m, which is as approximately three orders of magnitude above a typical high-velocity region local step in the clean trajectories. Particles with multiple flagged jumps were excluded because repeated discontinuities would dominate velocities and power spectra. For trajectories with a single flagged jump, the first flagged index was interpreted as the onset of non-physical return-to-origin decay, and subsequent samples were discarded. After filtering, particles with at least K=55 valid pre-decay timesteps were retained. The resulting filtered DMD set contained 404 all-tracks particles and 290 stuck-particle trajectories, for 694 retained trajectories. This filtering reduced the influence of discontinuous artifacts on derivative-based summaries, but it also biased the retained DMD set toward the most stable reconstructions.

The DMD reconstructions spanned 60 timesteps for the all-tracks group and 55 timesteps for the stuck-particle group. The diffusion samples were uniformly subsampled from $K_{\text{full}}=1024$ to K=55, with the same frame selection applied to the reference tracks. Under this mapping, the common frame spacing was taken as $\Delta t\approx 1.279\;\text{s}/54\approx 2.370\times 10^{-2}\;\text{s}$. Because the DMD export and the DDPM/reference tensor were generated through different pipelines, temporal diagnostics that depend on an absolute time axis, including autocorrelation lag and PSD frequency, should be interpreted primarily as frame-matched comparisons.

For speed, velocity components, and multi-lag increment norm, the reported comparison used Wasserstein-1 distance $W_{1}$, the Kolmogorov–Smirnov statistic $D_{\text{KS}}$, and energy distance Ramdas et al. (2017); Massey (1951); Székely and Rizzo (2013). The definitions and interpretation of these quantities were summarized in Methods section. Table 2 emphasizes $W_{1}$ and retains $D_{\text{KS}}$ as a secondary diagnostic. The KS p-values were reported as <0.05.

#### 3.5.2. Speed and component velocities

Speed and component velocities were computed from first-order position differences, ${\hat{v}}_{k}=\left(x_{k+1}-x_{k}\right)/\Delta t$, on the common filtered Geometry 1 frame grid. The mean speed across all particles and timesteps was 0.216 m s^−1^ for the reference trajectories, 0.222 m s^−1^ for guided DDPM, and 0.214 m s^−1^ for DMD. The DMD mean was close to the reference mean, but the distribution differed. Speed samples below 0.01 m s^−1^ accounted for 67.8% of DMD samples, compared with 72.5% for guided DDPM and 73.3% for the reference trajectories. The DMD 99^th^ percentile, 1.94 m s^−1^, exceeded the reference value (1.32 m s^−1^) and the guided DDPM value (1.36 m s^−1^) by approximately 45%. The reported speed $W_{1}$ values were 6.19 × 10^−2^ m s^−1^ for DMD and 6.59 × 10^−3^ m s^−1^ for guided DDPM, corresponding to a 9.4× lower speed-distribution distance for guided DDPM.

**Figure 8:**
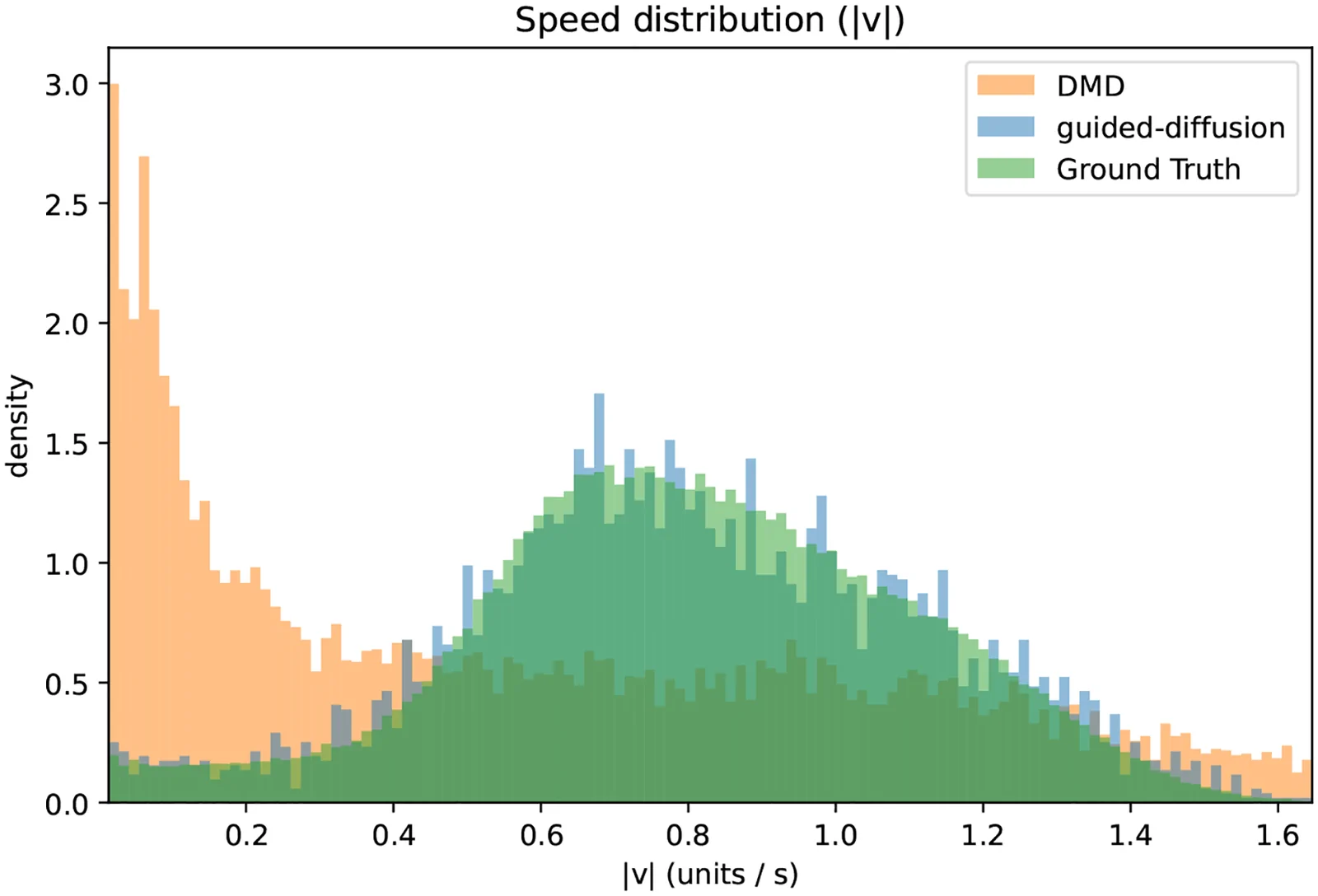
Speed distribution p(|v|) for guided DDPM (blue), filtered DMD (orange), and reference trajectories (green) in Geometry 1. The first histogram bin, which contains the near-zero stationary-particle plateau caused in part by last-value-held terminal segments, is suppressed so that the distribution above the plateau is visible. DMD has more mass in the plotted low-speed interval and a heavier upper tail in this filtered comparison.

**Figure 9:**
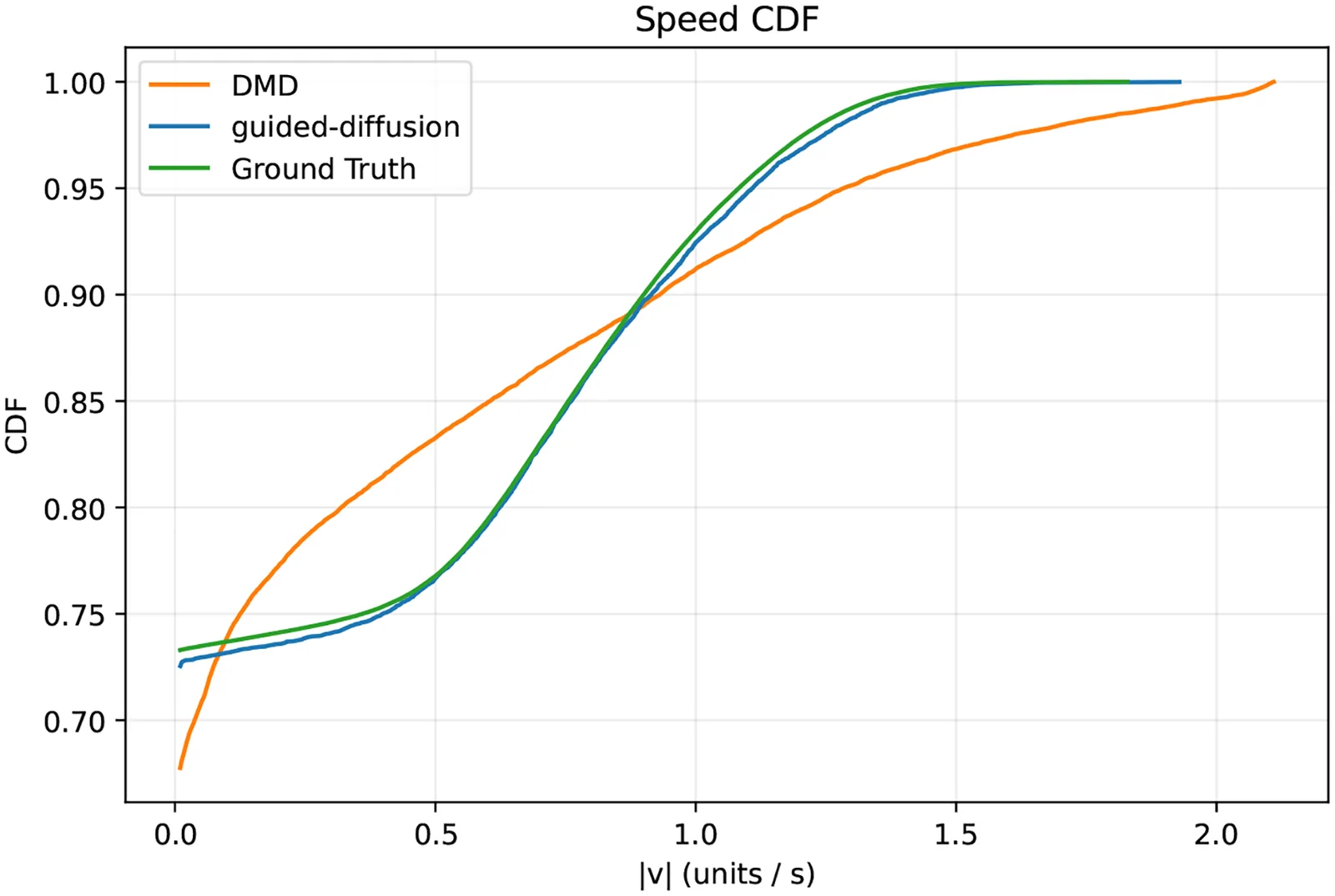
Empirical cumulative distribution functions of |v| for the filtered Geometry 1 comparison, with samples below 0.01 m s^−1^ omitted to show the dynamic range above the stationary component. The curves characterize pooled finite-difference speeds and should not be interpreted as terminal-event or deposition metrics.

Figures 8 and 9 show the corresponding distributions (note that the oversaturated near-zero bin was ommitted here and in the analysis). The guided DDPM and reference distributions have similar bulk locations, with a peak near $|v|\approx 0.7\;\text{m}{\;\text{s}}^{-1}$ and tapering above $|v|\approx 1.4\;\text{m}{\;\text{s}}^{-1}$. The DMD density distribution contains higher probability in the low-speed interval $|v|\in [0.0,0.4]\text{m}\;{\text{s}}^{-1}$ and a slower-decaying right tail. The empirical cumulative distributions show the same redistribution: the DMD curve lies to the left of the reference through part of the bulk distribution and then crosses into a heavier high-speed tail. Thus, confirmining that similar mean speeds do not imply similar speed distributions.

#### 3.5.3. Increment statistics across lags

A lag τ denotes the number of frames separating two particle positions. The multi-lag increment norm, $\|\Delta x(\tau ){\|}_{2}={\left\|x_{k+\tau }-x_{k}\right\|}_{2}$, measures the distance traveled over that frame lag. Short lags compare local step sizes, whereas longer lags compare accumulated branch-scale displacement and the temporal ordering of motion along the trajectory. This metric is useful because two trajectory sets can have similar instantaneous speed distributions while differing in how those speeds are arranged along each track.

**Figure 10:**
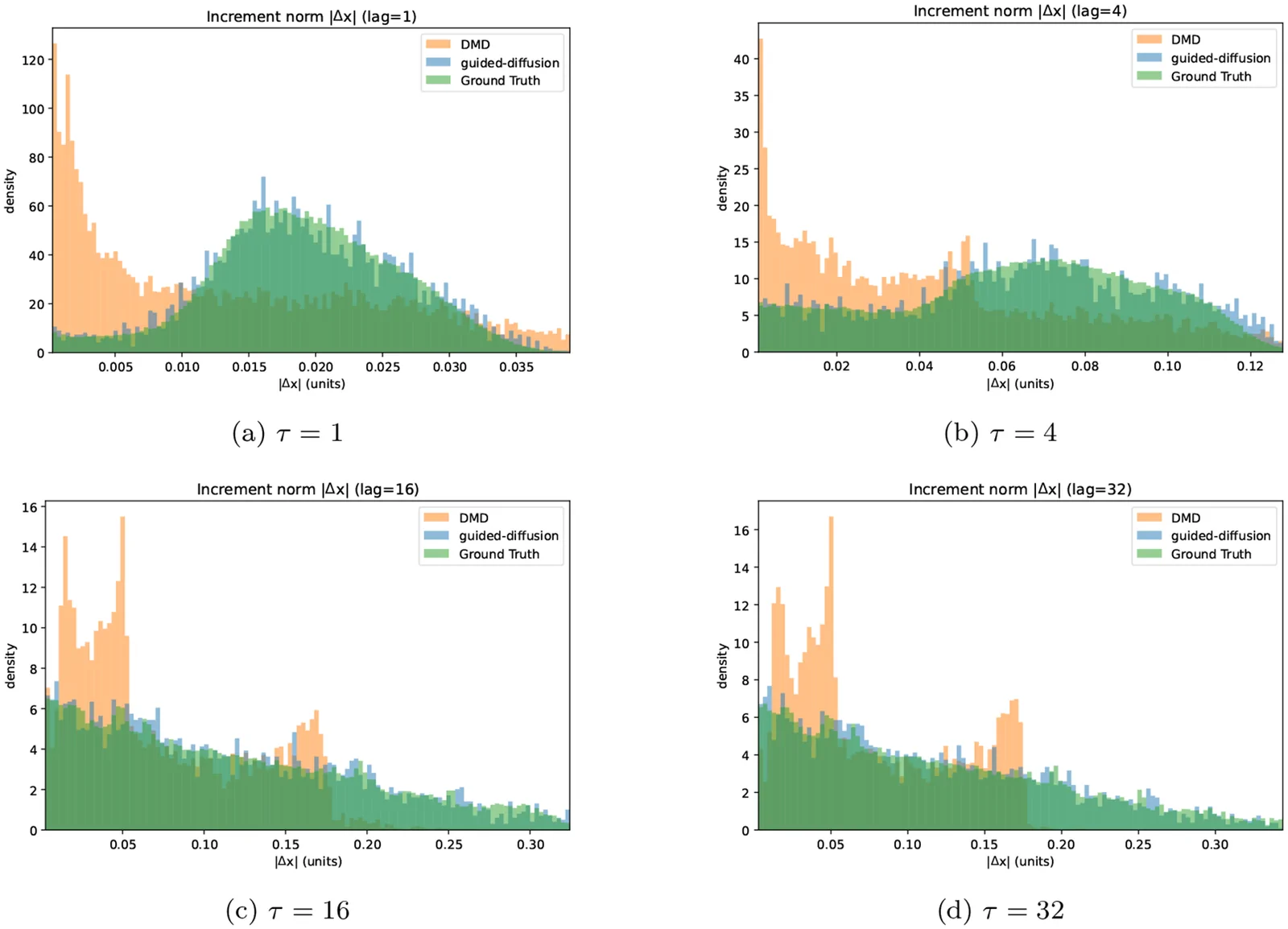
Increment-norm densities $p\left(\|\Delta x(\tau ){\|}_{2}\right)$ at four frame lags for guided DDPM (blue), filtered DMD (orange), and reference trajectories (green) in Geometry 1. The first bin is suppressed to display the range above the stationary component. The comparison is exploratory because the models represent different tasks and the DMD sample was filtered before derivative-based metrics were computed.

The reported analysis used τ∈{1,4,16,32}. Under the adopted effective time mapping, these lags correspond to approximately 0.024 s to 0.76 s, but the primary interpretation remains frame matched. Figure 10 reports the increment-norm densities (note, again the first bin suppressed). Across the four lags, the guided DDPM distributions are closer to the reference distributions than the filtered DMD distributions by the reported $W_{1}$ values in Table 2. The filtered DMD distributions develop narrow peaks at characteristic magnitudes, including approximately 0.04 m and 0.16 m, that are not apparent in the reference distributions. These peaks become more prominent at longer lags, consistent with repeated modal excursions in the filtered DMD reconstruction.

#### 3.5.4. Temporal correlations and spectra

The normalized speed autocorrelation, $R_{\left|v\right|}(\tau )$, measures how speed at one frame is correlated with speed at a later frame. It was included because the speed histogram alone does not determine whether speeds are placed in a similar temporal order along each particle path. For each trajectory, the reported analysis mean-centered the speed sequence, correlated it with a lagged copy, zero-offset normalized the result so that $R_{\left|v\right|}(0)=1$, and averaged over trajectories.

**Figure 11:**
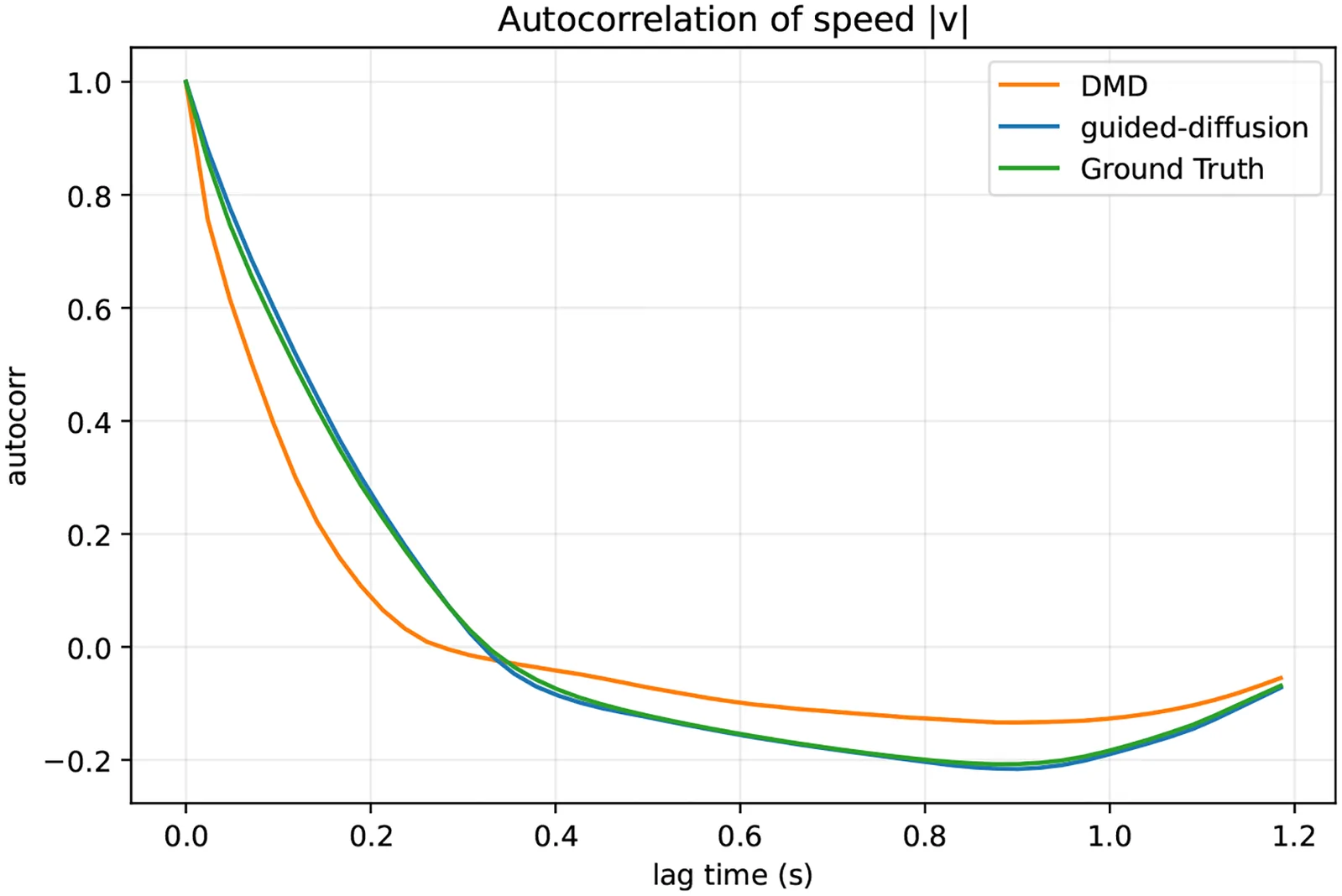
Autocorrelation of speed $R_{\left|v\right|}(\tau )$ for guided DDPM (blue), filtered DMD (orange), and reference trajectories (green) in Geometry 1. Lags are primarily interpreted in frames; conversions to seconds use the adopted effective mapping described in the text.

Figure 11 shows similar guided DDPM and reference autocorrelation curves in the source analysis. Under the adopted effective time mapping, both curves decay from 1 toward zero by approximately τ≈0.4s, reach a minimum of about −0.2 near τ≈0.9s, and then recover toward zero. The filtered DMD curve decays faster, with R(0.1)≈0.5 compared with approximately 0.7 for the reference, and reaches a shallower trough of approximately −0.13. These physical-time values depend on the adopted mapping and should be interpreted as frame-matched diagnostics rather than independently verified physical lag estimates.

The same temporal structure was evaluated spectrally using the one-sided power spectral density (PSD) of each velocity component. PSD decomposes the variance of a time series across frequency, and its relationship to the autocovariance function follows the Wiener–Khinchin theorem Zbilut and Marwan (2008). In this frame-matched analysis, lower frequencies correspond to slowly varying component motion along coherent airway paths, whereas higher frequencies correspond to rapid component-to-component fluctuations.

**Figure 12:**
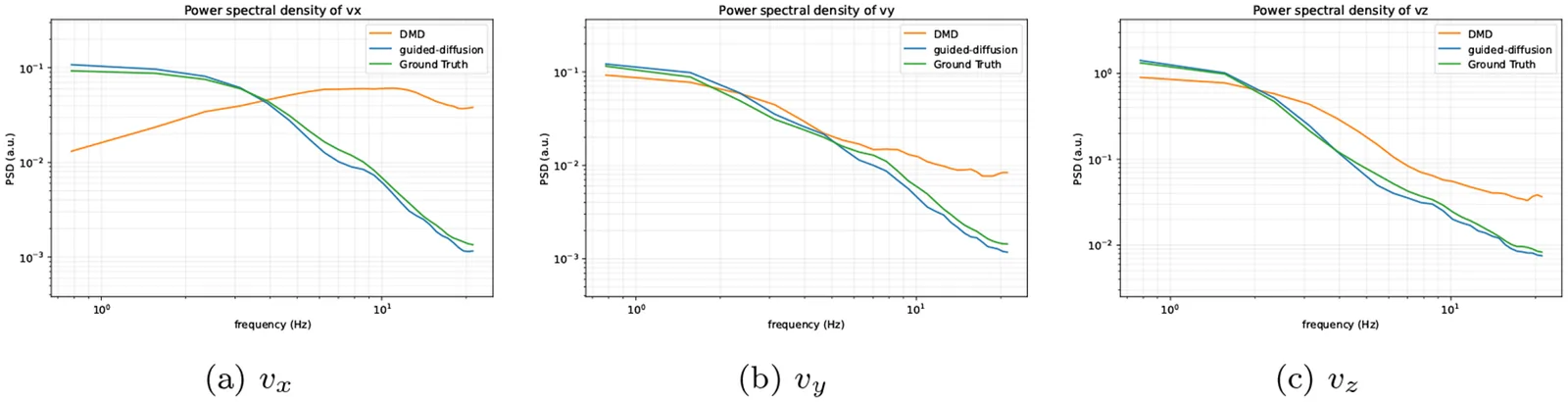
Mean one-sided PSD of each velocity component for guided DDPM (blue), filtered DMD (orange), and reference trajectories (green) in Geometry 1. Frequency values in hertz use the adopted effective frame-to-time mapping, so the spectral comparison should be interpreted primarily as a frame-matched diagnostic.

Figure 12 plots the mean PSD on log-log axes. The reference and guided DDPM spectra follow a decaying shape, with the reported PSD dropping by roughly two decades between f≈1Hz and the adopted Nyquist frequency $f_{\text{Ny}}\approx 21\;\text{Hz}$. The guided DDPM spectra were reported within approximately a factor of 1.5 of the reference across the plotted band for $v_{x}$ and $v_{y}$, and within a factor of 2 for $v_{z}$. The filtered DMD spectrum differs by remaining flatter or rising with frequency, most visibly for $v_{x}$. At the adopted f≈10Hz value, the DMD PSD for $v_{x}$ exceeded the reference by about an order of magnitude, while near f≈1Hz it was roughly an order of magnitude lower.

#### 3.5.5. Summary of distributional metrics

Table 2 consolidates the reported Wasserstein distances and KS statistics for the metrics considered in this analysis. Across the selected diagnostics, the guided DDPM had lower reported $W_{1}$ values than the filtered DMD reconstruction. The DMD/guided ratios ranged from 2.1 for $v_{z}$ to 16.0 for the τ=16 increment norm. Because the models represent different tasks and the DMD output was heavily filtered, this comparison should be interpreted as an exploratory contrast rather than a definitive ranking of surrogate models.

#### 3.5.6. DMD and DDPM limitations

The comparison across Figure 7 to Figure 12 and Table 2 led to a consistent interpretation. Guided DDPM reproduced the ground-truth speed distribution, component-velocity distributions, multi-lag increment statistics, autocorrelation, and PSD within the resolution of these diagnostics. DMD reproduced the gross mean speed scale, but the matching mean masked distributional differences. DMD combined excess slow-particle probability with an overly heavy high-speed tail and modal peaks in long-lag displacement.

These differences reflected known limitations of DMD reduced-order reconstructions. First, the learned DMD operator is low-rank and linear in the selected observable space. This structure is useful for coherent dynamics, but it is not designed to represent intermittent, particle-specific events such as wall interactions, branch transitions, sharp geometric turns, or stochastic submicron forcing. Second, the DMD reconstruction is unconstrained by the airway surface. Unlike the guided DDPM sampler, it does not evaluate a signed distance function or apply a boundary penalty during generation. Third, DMD is fitted to a fixed state dimension and coordinate system. A new airway geometry would require new high-fidelity snapshots and a new operator, or a validated state-mapping procedure, rather than direct evaluation of the existing DMD model. Fourth, DMD is deterministic after the operator and amplitudes are specified, so it does not naturally sample the full conditional distribution of plausible Lagrangian tracks.

The jump-filtering step for the DMD data was deemed fair for numerical comparison, but it introduced its own limitation. Removing particles with multiple discontinuities and truncating trajectories at the first return-to-origin event prevented non-physical jumps from dominating finite-difference velocities and PSD values. However, the retained 694-particle DMD ensemble represented only the most stable portion of the reconstruction. The 404 retained all-tracks particles corresponded to 2.3% of the 17 840 all-tracks output. The fact that DMD still trailed guided DDPM after this favorable filtering indicated that the remaining discrepancy was not solely an artifact of unfiltered jump events.

The PSD comparison in Figure 12 shown that velocity-component spectra contained more low-frequency than high-frequency power, reflecting persistent translation through the airway with smaller high-frequency fluctuations. Guided DDPM reproduced this roll-off. DMD produced a flatter or rising spectrum, indicating underrepresentation of slow coherent motion and overrepresentation of high-frequency oscillatory content. The faster DMD autocorrelation decay in Figure 11 was the corresponding time-domain manifestation of the same spectral imbalance.

For downstream tasks that depend on single-particle increments (including deposition-site localization, residence-time analysis, and transport-coefficient estimation) the DMD particle-track surrogate was therefore less reliable than the guided DDPM model in this comparison. The τ=16 increment distance, which lies in the lag range relevant to branch-scale transport over the reduced 55-frame window, was 16× larger for DMD than for guided DDPM. The guided DDPM model also retained a practical advantage that DMD in its current version did not: once trained, it could be conditioned on a new airway representation, whereas a fitted DMD operator could not be transferred to a new anatomical mesh without additional simulation and operator identification. On the trained/fitted geometry, the guided DDPM model reproduced single-particle speed, component-velocity, multi-lag increment, autocorrelation, and PSD statistics more accurately than the DMD reconstruction, which one may contradict that this analysis may not be fair due to using a geometry that was on the training dataset, but likewise was for the DMD case.

## 4. Discussion

Across geometries, the guided denoising diffusion probabilistic model closely matched ground-truth mean kinematics but tends to mildly smooth extreme events. In Table 1, speed mean was typically within a few percent of ground truth, while speed p99 and acceleration p99 were consistently lower than ground truth in most cases, indicating slight under-representation of the highest-speed and highest-acceleration events. This behavior demonstrated consistent with denoising-based generative models where guidance and denoising reduced tail variability when the training distribution was limited.

Wasserstein distances between speed distributions were small (on the order of 10^−3^ to 10^−2^), indicating that the guided DDPM model recovered the overall speed distribution shape well, even when some tail mass is slightly reduced.

Geometry 4 shown the smallest Wasserstein distance ($W_{1}\approx 0.00135$), and the kinematic metrics were extremely close (speed p99 differed negligibly). On the other hand, Geometry 6 standed out due to the mean speed and mean path length being notably lower than ground truth, and $W_{1}$ was among the largest, indicating that the generated ensemble departed the most from the ground-truth statistics in this case. This trend was consistent with the qualitative behavior observed in guidance comparisons in Figure 4, for difficult geometries, guidance can improve feasibility but may also bias trajectories toward dominant pathways, effectively having the effect of reducing path length and suppressing high-speed excursions.

These results should be interpreted in light of the training regime as well, although each CFPD simulation yielded many trajectories (rich sampling of the kinematic distribution), the number of distinct simulated geometries was relatively small. Diffusion models robustly learn trajectory statistics given sufficient samples, but geometry-specific generalization is fundamentally constrained by the diversity of airway shapes seen during training. Therefore, the observed performance is encouraging for kinematic representation of the particle paths and distributional recovery, while the feasibility and generalization limitations point directly to dataset expansion (more geometries) and improved geometry alignment for signed distance function evaluation as high-impact next steps.

Beyond geometry, additional conditioning variables can be appended in future ablations, e.g., particle diameter $d_{p}$, Stokes number St, inlet flow rate parameters $\left(Q_{\text{max}},T\right)$, or event-type priors (deposition vs escape). These can enter the model as feature, embeddings added to $e_{\text{cond}}$ or via feature-wise linear modulation (FiLM).

The qualitative analysis demonstrated that diffusion-based generative modeling remains a practical and effective paradigm for synthesizing Lagrangian particle trajectories conditioned on subject-specific airway geometry. Two aspects of the proposed framework were particularly important and are detailed below.

First, geometry conditioning via a variational autoencoder enabled non-trivial generalization to geometries not encountered during training. The number of distinct anatomies available for training and testing was only nine. This imbalance created a fundamental bottleneck for learning geometry-specific behaviors, especially in distal branches where inter-subject variability was higher. In this context, the observed qualitative agreement on unseen geometries should be interpreted as interpolation within a sparsely sampled geometry manifold rather than broad robustness to arbitrary airway anatomy. Nevertheless, the ability to produce plausible trajectories on held-out cases indicated that the latent geometry representation captured meaningful anatomical factors for conditioning the diffusion model.

Second, SDF guidance played a critical role during inference by improving geometric plausibility. Unguided sampling drifted into non-physical regions, yielding trajectories that violated airway boundaries or form implausible bundles not aligned with the lumen. Incorporating guidance consistently suppressed outliers and increased general alignment with airway topology, particularly in the trachea and main bronchi. At the same time, the results suggested a trade-off. Stronger guidance reduced sample diversity by concentrating trajectories in dominant pathways and under-representing peripheral branches, which as a consequence it will demand a larger number of trajectory samples when are synthetically generated, as opposed to CFPD generated ones. This behavior was consistent with a guidance mechanism that biases sampling toward geometric feasibility. In practice, the guidance weight should therefore be treated as a tunable parameter that balances constraint satisfaction against faithful reproduction of trajectory variability.

Another outcome was that the guided DDPM model was able to recover velocity magnitude statistics with good agreement to CFPD-derived results, as evidenced by the cumulative distribution functions and histograms. This finding supports the broader expectation that diffusion models are well-suited to learning complex, multimodal distributions without the training instabilities and mode collapse commonly associated with adversarial objectives Liu et al. (2020); Dhariwal and Nichol (2021). Even under limited geometry diversity, the model benefited from the large number of trajectory samples per geometry and learned robust marginal kinematic statistics.

### 4.1. Limitations

Several limitations must be emphasized to frame the current contributions appropriately and to guide future work.

The most significant limitation was the small number of geometries with particle-track simulations. With only nine simulated anatomies, the model could not fully learn the breadth of anatomical variability presented in population-scale airway datasets. As a consequence, performance degraded for some of the out-of-distribution geometries, where the model hallucinated trajectory patterns that were not physically meaningful (e.g., incorrect branch volumetric occupation or implausible concentration patterns). This limitation was structural and not easy to solve, since improving generalization will require increasing the number of simulated geometries, which requires extra thousands of CPU-hours.

On the residual boundary violations, although signed distance function guidance improves feasibility, some generated trajectories still crossed physical boundaries in challenging cases (e.g., near complex bifurcations, or in thin distal branches). This indicated that guidance alone on the predicted mean for denoising, was not a complete guarantee of constraint satisfaction, especially if the conditioning latent places the sampler near poorly supported regions of the training distribution. Guidance can reduce non-physical outliers but may also reduce diversity in distal airways and slightly bias the distribution of rare events. This may be especially relevant for applications where capturing tail behavior (rare high-speed jets, near-wall residence events, or extreme accelerations) is important. Moreover, the current framework conditions primarily on geometry (via the variational autoencoder latent) and used a fixed or implicitly learned particle-size distribution from the training data. Realistic aerosol transport for any material depends strongly on particle diameter and density. Extending the model to incorporate or conditioning particle size (either by providing the diameter as an additional conditioning variable for each trajectory or by modeling mixtures of size-resolved conditional distributions) is a fundamental next step. This would enable inference for arbitrary size distributions and would support patient- and scenario-specific prediction at inference time.

Finally, while diffusion-based inference is fast once trained, expanding the training set with additional geometries or particle distributions requires more high-fidelity CFPD simulations and therefore more computational time. This creates a clear roadmap for improvement, but also highlights the need for careful experimental design and potentially hybrid data strategies (e.g., combining a smaller number of high-fidelity simulations with additional lower-cost simulations, likely one-way coupled, or using synthetic geometry augmentation validated against physics).

### 4.2. Future work

Future work should extend the present particle-trajectory formulation in several directions. A first task is explicit modeling and evaluation of active, deposited, and escaped terminal states, rather than representing terminal behavior only through position sequences and last-value hold. Such a model would require event-aware training targets and separate validation of terminal-event accuracy.

A second gap to address is conditional control over variables that were fixed or implicit in the current training distribution. Particle diameter, injection state, particle-size distribution, and breathing waveform parameters should be represented as explicit conditioning variables before the model is used for scenario-specific trajectory generation. These additions would need validation against reference trajectories generated under corresponding simulation regimes.

Larger and more diverse anatomy-level datasets would be needed as well for broader validation. Additional simulated geometries would support more informative train/validation/test splits, more rigorous assessment of anatomy-conditioned generation, and better characterization of failure modes in distal branches. Future model development should also investigate multiscale or local geometry conditioning. A single global occupancy embedding may be insufficient to represent local branch structure, distal airway features, and bifurcation-scale constraints. Local surface, centerline, branch, or patch-based conditioning could be evaluated prospectively, provided that the resulting conditioning variables are defined reproducibly and tested against reference trajectories.

Lastly, additional work is needed to verify SDF and coordinate consistency and to evaluate stronger constrained-sampling methods. Future studies should report quantitative containment diagnostics, guidance-scale sensitivity, and failure cases near thin or high-curvature structures. Direct evaluation of deposition outcomes should be treated as a separate downstream validation step rather than assumed from trajectory-shape or kinematic agreement. Uncertainty, sensitivity, and ablation analyses would further clarify which parts of the framework drive the reported behavior.

## 5. Conclusions

This work introduced a geometry-conditioned, guided DDPM for generating Lagrangian particle trajectories in subject-specific human airway geometries. Motivated by the need for fast, anatomy-aware particle tracking for aerosol deposition analysis, inhaled drug delivery optimization, and radiation research, the proposed approach addresses critical limitations of CFPD simulations by learning a direct generative surrogate from simulation-derived trajectories. The framework combined a variational autoencoder geometry latent for conditioning with SDF guidance during inference to improve geometric feasibility and reduce non-physical wall-penetrating samples.

Across qualitative trajectory comparisons and quantitative analyses, the guided diffusion model produced physically plausible ensembles that matched key statistical properties of ground-truth trajectories, including on geometries not seen during training. These results are particularly encouraging given the extremely limited number of simulated geometries available. Remaining limitations, including occasional boundary violations and reduced robustness for out-of-distribution anatomies, highlight the importance of expanding the diversity of simulated geometries and enriching conditioning variables (such as particle size and breathing rates) in future studies. Overall, the proposed method demonstrates a viable pathway toward rapid, geometryaware Lagrangian particle trajectory generation, bridging the gap between high-fidelity simulation and practical subject-specific deployment.

## Figures and Tables

**Table 1:** Summary of trajectory-derived kinematic metrics and speeddistribution distance. For each listed geometry, reference and guided DDPM values are reported for mean acceleration magnitude, 99^th^ percentile acceleration magnitude, mean path length, mean speed magnitude, 99^th^ percentile speed magnitude, speed standard deviation, and Wasserstein-1 distance between pooled speed distributions. Acceleration is in m s^−2^, path length is in m, and speed quantities and speed $W_{1}$ are in m s^−1^. These metrics characterize generated position sequences and their finite-difference kinematics; they do not assess deposition outcomes.

Geometry	Set	Accel. mean (ms^−2^)	Accel. p99 (ms^−2^)	Path length (m)	Speed mean (ms^−2^)	Speed p99 ($ms^{-1}$)	Speed std (ms^−2^)	Speed $W_{1}$ (ms^−2^)
1	Reference	55.33	568.3	0.2822	0.2207	1.680	0.4291	–
	Guided DDPM	54.77	518.9	0.2798	0.2188	1.528	0.4042	0.0181
2	Reference	54.86	518.1	0.2781	0.2175	1.520	0.3967	–
	Guided DDPM	54.92	505.1	0.2761	0.2159	1.478	0.3877	0.00618
3	Reference	64.73	561.1	0.3113	0.2435	1.653	0.4578	–
	Guided DDPM	60.33	524.3	0.3079	0.2408	1.550	0.4034	0.0209
4	Reference	45.44	535.3	0.2273	0.1778	1.599	0.3853	–
	Guided DDPM	45.01	531.8	0.2259	0.1767	1.597	0.3837	0.00135
6	Reference	58.44	550.7	0.2995	0.2342	1.602	0.4247	–
	Guided DDPM	53.95	532.0	0.2717	0.2125	1.546	0.4028	0.0217
7	Reference	55.15	543.4	0.2826	0.2210	1.604	0.4171	–
	Guided DDPM	53.68	523.0	0.2773	0.2168	1.528	0.4053	0.00509

**Table 2:** Distributional distances for the exploratory, jump-filtered Geometry 1 comparison. “Guided” refers to the guided DDPM model, and “DMD” refers to the filtered DMD reconstruction. $W_{1}$ is the Wasserstein-1 distance, with units determined by the row variable. The ratio is DMD divided by guided DDPM. The KS statistic is reported for completeness, but its values were strongly affected by the near-zero stationary component and should not be interpreted as a standalone practical effect size.

Statistic	$W_{1}$ guided	$W_{1}$ DMD	Ratio DMD/guided	$D_{KS}$ (guided / DMD)
$|v|\left[\text{m}{\text{s}}^{-1}\right]$	6.59 × 10^−3^	6.19 × 10^−2^	9.4	0.732 / 0.732
$v_{x}\left[\;\text{m}{\text{s}}^{-1}\right]$	4.40 × 10^−3^	2.30 × 10^−2^	5.2	0.368 / 0.391
$v_{y}\left[\;\text{m}{\text{s}}^{-1}\right]$	4.95 × 10^−3^	1.29 × 10^−2^	2.6	0.371 / 0.386
$v_{z}\left[\;\text{m}{\text{s}}^{-1}\right]$	1.98 × 10^−2^	4.21 × 10^−2^	2.1	0.375 / 0.387
$\|\Delta x{\|}_{2}[\text{m}],\tau =1$	1.56 × 10^−4^	1.47 × 10^−3^	9.4	0.732 / 0.732
$\|\Delta x{\|}_{2}[\text{m}],\tau =4$	7.58 × 10^−4^	4.12 × 10^−3^	5.4	0.717 / 0.717
$\|\Delta x{\|}_{2}[\text{m}],\tau =16$	8.65 × 10^−4^	1.38 × 10^−2^	16.0	0.629 / 0.629
$\|\Delta x{\|}_{2}[\text{m}],\tau =32$	1.79 × 10^−3^	2.41 × 10^−2^	13.4	0.381 / 0.381

## Data Availability

A permanent repository for CFPD ready geometries is available at dx.doi.org/10.6084/m9.figshare.24787773Bartol et al. (2024a). All the other relevant data are available from the corresponding author upon reasonable request.

## Code Availability

The code can be found at https://github.com/ignaciobartol/airway-lagr-diffusion
